# Genomic locus of lncRNA-*Gm26793* forms an inter-chromosomal interaction with *Cubn* to ensure proper stem cell differentiation in vitro and in vivo

**DOI:** 10.1038/s41421-025-00805-0

**Published:** 2025-06-03

**Authors:** Zhiwen Liu, Xin Wan, Jiehui Chen, Yongjian Ma, Yonggao Fu, Yingying Chen, Mingzhu Wen, Yun Yang, Yun Qian, Yong Zhang, Dahai Zhu, Jinsong Li, Naihe Jing, Xianfa Yang

**Affiliations:** 1 Guangzhou National Laboratory, Guangzhou, Guangdong China; 2 State Key Laboratory of Medical Molecular Biology, Institute of Basic Medical Sciences, Chinese Academy of Medical Sciences and School of Basic Medicine, Peking Union Medical College, Beijing, China; 3 State Key Laboratory of Cell Biology, Shanghai Key Laboratory of Molecular Andrology, CAS Center for Excellence in Molecular Cell Science, Shanghai Institute of Biochemistry and Cell Biology, University of Chinese Academy of Sciences, Chinese Academy of Sciences, Shanghai, China; 4 Bioland Laboratory (Guangzhou Regenerative Medicine and Health Guangdong Laboratory), Guangzhou, Guangdong China

**Keywords:** Embryonic stem cells, Long non-coding RNAs, Chromatin structure

## Abstract

Inter-chromosomal interactions play a crucial role in 3D genome organization, yet the organizational principles and functional significances remain elusive. In general, lncRNA loci and transcripts are frequently associated with transcriptional programs modulated by long-range chromatin interactions. Here, we identified a novel lncRNA named *Gm26793*, which is abundantly distributed in the primitive streak and mesodermal cells of embryonic day 7.5 mouse gastrula. Through genetic ablation of *Gm26793*, we observed a preferential responsiveness to primitive endoderm lineage during stem cell differentiation, as well as enhanced occurrence of transient and degenerative state cells in early mouse embryos when the cell fate segregates between epiblast and primitive endoderm. Mechanistically, we revealed that the genomic locus of *Gm26793*, rather than the lncRNA transcript or adjacent gene, governs the cell fate preference towards primitive endoderm. Concretely, *Gm26793* locus (Chromosome 7) forms an inter-chromosomal molecular lock with *Cubn* (Chromosome 2) via CTCF, restraining the expression of *Cubn* and maintaining a natural epigenetic landscape, thus ensuring the proper lineage specification in vitro and in vivo. Overall, our study provides a clear paradigm that inter-chromosomal interaction collaborates with architectural factors to stabilize nuclear conformation and guarantee faithful gene expression during stem cell differentiation and mammalian embryogenesis.

## Introduction

Mammalian genomes encode tens of thousands of non-coding dark matter, such as lncRNA genes, which have been found to execute crucial biological functions^1–3^. However, the precise determination of specific functional lncRNAs usually tends to be blind and labor-intensive until the coming era of high-throughput sequencing and efficient genomic editing. Pre-screening through exploration of lncRNA abundance in specific biological tissues, especially across spatial-temporal embryo developmental transcriptomic atlas or disease-related transcriptome reference, could largely facilitate the identification of vital lncRNAs with biological significance^4–7^. Pioneering mechanistic studies have reported that the lncRNA genes can modulate chromatin structures and regulate the expression of local or distal genes, frequently through the act of transcription and genomic loci^8–12^. Nevertheless, massive gaps still exist in the understanding of the regulatory purposes of ubiquitous lncRNAs and how they differ from the established regulatory network mediated by coding genes. Furthermore, the extent to which genetic removal of diverse lncRNAs can result in physiologically relevant phenotypes remains unclear.

In mammalian cells, the linear sequence of the genome is hierarchically organized into distinct chromosome territories, A/B compartments, topologically associating domains (TADs) and chromatin loops^13–16^. These structural units ensure the overall genome stability as well as maintain the relative plasticity of chromatin interaction against specific physiological stimuli^17–21^. Among these structural units, the chromatin loops formed via chromatin folding seem to be of the highest flexibility and usually exhibit dramatic dynamics of loop switching upon the stimuli of certain differentiation signals or chemical treatment^22–25^. As revealed by the typical chromatin interaction capture technology, most of the chromatin loops seem to exist between *cis*-acting anchor sites within merely one chromosome^26–29^. However, for most biological processes, the genome set usually acts as an entirety and exhibits a coordinated change of conformation upon extracellular stimulation. Thus, how the cells can harmonize the entire set of chromosomes within the nuclei remains largely unknown^30–34^. The occurrence of direct inter-chromosomal interactions or *trans*-acting contacts provides one potential strategy for the coordination of chromatin conformation in response to certain stimuli^35–40^. For example, major inter-chromosomal hubs have been reported to arrange around the nuclear bodies and also correspond to RNA polymerase II transcriptional status^35^. Meanwhile, the complex choreography of olfactory receptor genes, which are located across several different chromosomes, involves frequent inter-chromosomal interactions in the form of the “olfactosome” to determine specific olfactory receptor genes’ expression in sensory neurons^41,42^. However, whether the inter-chromosomal interactions also exist and execute critical biological functions during early development remains largely unexplored.

The CCCTC-binding factor, CTCF, is a major organizer in orchestrating the chromatin interactions within individual chromosomes and between different chromosomes^43–45^. In the prevailing model of loop-extrusion, the cohesin complex can form a “ring” to capture a chromatin loop and slide through the chromatin until it encounters a pair of convergent CTCF-binding sites^46–48^. The boundary areas of chromatin loops within individual chromosomes are generally found to be crucial regulatory elements (such as enhancers, promoters, silencers, and insulators), which are tightly related to gene expression regulation^49–54^. Therefore, the binding of CTCF engages and determines the coordination of distinct genomic loci, thus ensuring the normal gene expression landscape and providing a proper cellular homeostasis^55–58^. Emerging studies implicate that CTCF participates in the formation of inter-chromosomal contacts^59–61^, whereas the specific functional properties of CTCF in regulating inter-chromosomal interaction await interpretation.

In this study, through systematic analyses of the established mouse spatial transcriptome atlas, we identified a lncRNA gene, *Gm26793*, specifically expressed in the primitive streak and mesoderm tissues of the E7.5 embryo, and found that genetic elimination of *Gm26793* leads to the aberrant upregulation of primitive endoderm genes in vitro, and causes developmental arrest during the cell fate segregation between epiblast and primitive endoderm in vivo. Molecularly, *Gm26793* (Chromosome 7) could form inter-chromosomal interaction with *Cubn* (Chromosome 2) through the genomic locus, but independent of the transcript and adjacent genes. This specific inter-chromosomal interaction functions as a molecular lock that limits the expression of *Cubn*, sustaining the appropriate epigenetic modification and differentiation capacity of mouse embryonic stem cells (mESCs). Additionally, akin to the secure fastening of a lock, the binding of CTCF can reinforce the linkage of chromatin interactions.

## Results

### The identification of functional lncRNA-*Gm26793* based on the spatial transcriptome atlas of the mouse gastrula

**Fig. 1:**
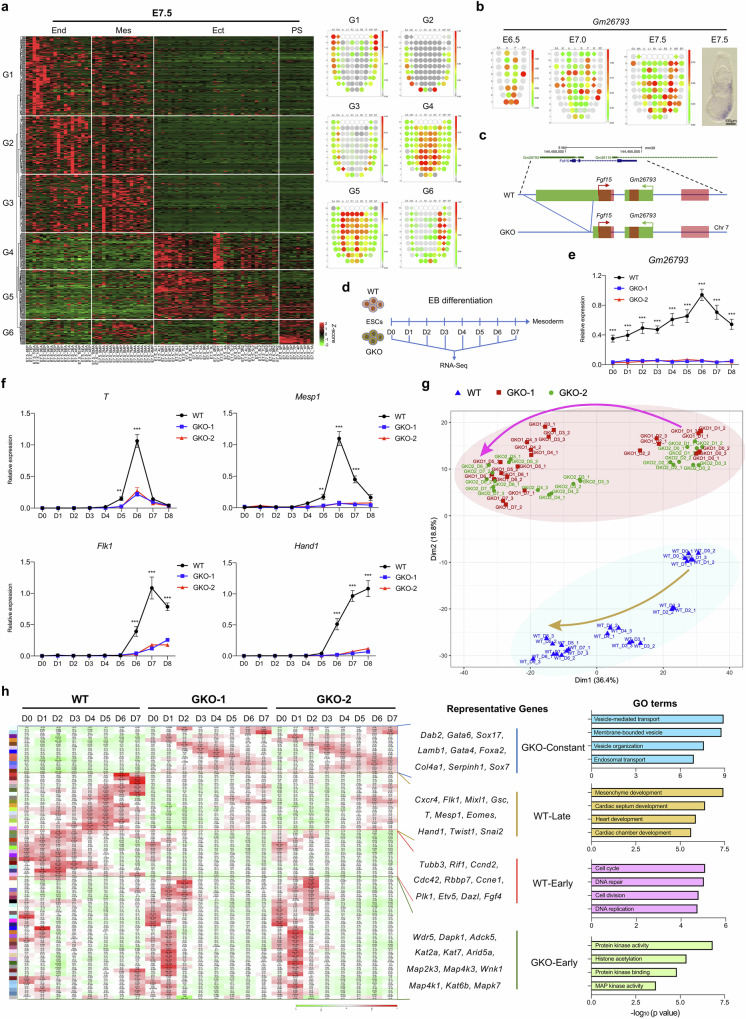
The identification of lncRNA-*Gm26793* distribution in the gastrula and its functional significance during mESCs differentiation. **a** DELs across ectoderm, mesoderm, endoderm, and primitive streak regions in the E7.5 embryo. The average expression pattern of each gene group is presented on the right panel as corn plots. End endoderm, Mes mesoderm, Ect ectoderm, PS primitive streak, A anterior ectoderm, P posterior ectoderm, L1 anterior left lateral ectoderm, R1 anterior right lateral ectoderm, L2 posterior left lateral ectoderm, R2 posterior right lateral ectoderm, EA anterior endoderm, EP posterior endoderm, MA anterior mesoderm, MP posterior mesoderm. **b** The spatial distribution of *Gm26793* transcript in the mouse gastrula. Experimental validation of *Gm26793* expression through whole-mount in situ hybridization was also shown. **c** Schematic diagram showing the strategy for the genomic knockout of *Gm26793*. **d** The workflow depicts the stem cell differentiation program and sampling timepoints for WT and *Gm26793* knockout cells. **e** qPCR (*n* = 3) analyses determining the relative expression changes of the *Gm26793* transcript during spontaneous differentiation. **f** Time-course expression profiles of mesoderm-related genes during WT and GKO EB differentiation measured by qPCR. **g** PCA analyses of EB differentiated samples show distinct clustering patterns and differentiation trajectories of WT and GKO mESCs. The major differentiation trajectories of WT and GKO mESCs are highlighted in the arrow lines. **h** Heatmap showing stage-specific co-expression gene modules and their correlation to differentiation days. Each row corresponds to a co-expression module. The correlation coefficients and *P* values are shown in each square. Representative genes and top GO terms of the four combined module categories are listed on the right panel. Data are shown as means ± SEM. Two-way ANOVA analysis with Tukey’s test is used in **e** and **f**; **P < 0.01, ***P < 0.001; *n* represents biologically independent replicates.

In this study, through in-depth analyses of our recently established spatial transcriptomic atlas of mouse gastrula^62,63^, we obtained numerous region-specifically expressed lncRNAs in embryo samples ranging from early-streak stage (E6.5), mid-streak stage (E7.0), to late-streak stage (E7.5) (Fig. 1a; Supplementary Fig. S1a–c and Table S1). Generally, we found that lncRNAs could form distinct expression patterns in the gastrula (Fig. 1a; Supplementary Fig. S1b–f), which is in accordance with the germ layer-related spatial location during mouse gastrulation^64,65^. To specify, based on the differentially expressed lncRNAs (DELs) during gastrulation, three gene groups could be identified with endoderm (End)-specific (G1, G2) or epiblast (Epi)-specific (G3) distribution (Supplementary Fig. S1b) for E6.5 embryos. As to the E7.0 embryo, along with the emergence of mesoderm tissues, two new gene groups of lncRNAs (G1, G2) can be identified with mesoderm-specific abundance (Supplementary Fig. S1c). When embryos developed to the E7.5 stage, six gene groups (G1, G2, G3, G4, G5, G6) could be identified (Fig. 1a). In contrast to embryos of the E6.5 and E7.0 stages, primitive streak cells in the E7.5 stage exhibit specific enrichment of certain lncRNAs, which are clustered into G6 group. Further investigation of G6 group-related lncRNAs revealed that these lncRNAs are highly expressed in the primitive streak, but gradually down-regulated in mesoderm tissues and largely absent in endoderm tissues and ectoderm tissues (Fig. 1a). Given that mesoderm and definitive endoderm cells are mostly derived from the primitive streak through epithelial-mesenchymal transition during gastrulation^66^, genes harbored in the G6 group may implicate potential biological importance during mesoderm and endoderm development.

Amongst the spatial-specific G6 lncRNAs, we found that one lncRNA named *Gm26793*, which shows partial genomic overlapping with the protein-coding gene *Fgf15*, exhibits high enrichment in the primitive streak region of E7.5 embryos (Fig. 1b, c). To investigate the biological significance of *Gm26793*, we took advantage of the CRISPR-Cas9 system to genetically delete the second exon of *Gm26793* (~1.5 kb) in mESCs, and retain the integrity of *Fgf15* transcripts (Fig. 1c; Supplementary Fig. S2a–c). Two biological replicates with genetic *Gm26793* knockout were prepared, and the resulting knockout embryonic stem cells were named GKO cells. Examination of the pluripotent marker expression as well as cellular morphology in GKO cells revealed that GKO cells still maintain comparable expression levels of key pluripotent markers in mESCs, such as *Oct4*, *Nanog*, and *Klf4* (Supplementary Fig. S2d, e), and the morphology of GKO clones is relatively loosely compacted in comparison with the tightly-organized wild-type (WT) mESCs (Supplementary Fig. S2e, f).

### The developmental potency towards mesoderm lineage is largely compromised in GKO cells in vitro

To determine the developmental potencies of GKO cells, we first subjected both WT and GKO cells to spontaneous differentiation in 10% fetal bovine serum (FBS) medium (Fig. 1d; Supplementary Fig. S2g)^67^. In accordance with in vivo transcriptomic data inferred from the gastrulation atlas, the expression level of *Gm26793* was gradually elevated and peaked at D6 of differentiation (Fig. 1e), when the cells reached a mesodermal state. Concomitantly, we found that the mesodermal cell markers, such as *T* and *Mesp1*, were largely abolished in GKO cells (Fig. 1f). To systematically assess the effects of *Gm26793* knockout, we collected the time-series bulk-cell transcriptomic data of both WT and GKO cells during this process. By performing principal component analysis (PCA), we found that the transcriptome differences between WT and GKO cells already exist at the embryonic stem cell stage (D0), and gradually widen along with differentiation from D0 to D7 (Fig. 1g). Next, we applied weighted gene co-expression network analysis (WGCNA)^68^ to evaluate the time-series transcriptomic distinctions between WT and GKO cells during spontaneous embryoid body (EB) formation. As shown in Fig. 1h, four temporal gene module categories with distinct stage- or sample-specific patterns can be identified, which can be named as WT-Early, WT-Late, GKO-Early, and GKO-constant, respectively (Supplementary Table S2). Gene ontology (GO) analyses further identified that genes in the WT-late category, which were less expressed in GKO samples, were highly related to mesoderm development. qPCR analyses and immunostaining reconfirmed the absence of transcript and protein levels of mesodermal genes in GKO cells during EB differentiation (Fig. 1f; Supplementary Fig. S2h, i). By contrast, endoderm-related genes, such as *Gata6*, *Sox17*, and *Sox7*, and relevant biological processes, were exclusively enriched in the GKO-constant category (Fig. 1h; Supplementary Fig. S3a). Overall, these results indicate that *Gm26793* knockout severely impedes the spontaneous differentiation of mESCs by down-regulating mesodermal genes and up-regulating endodermal genes.

### The developmental capacity towards primitive endoderm fate is abnormally enhanced in GKO cells in vitro

**Fig. 2:**
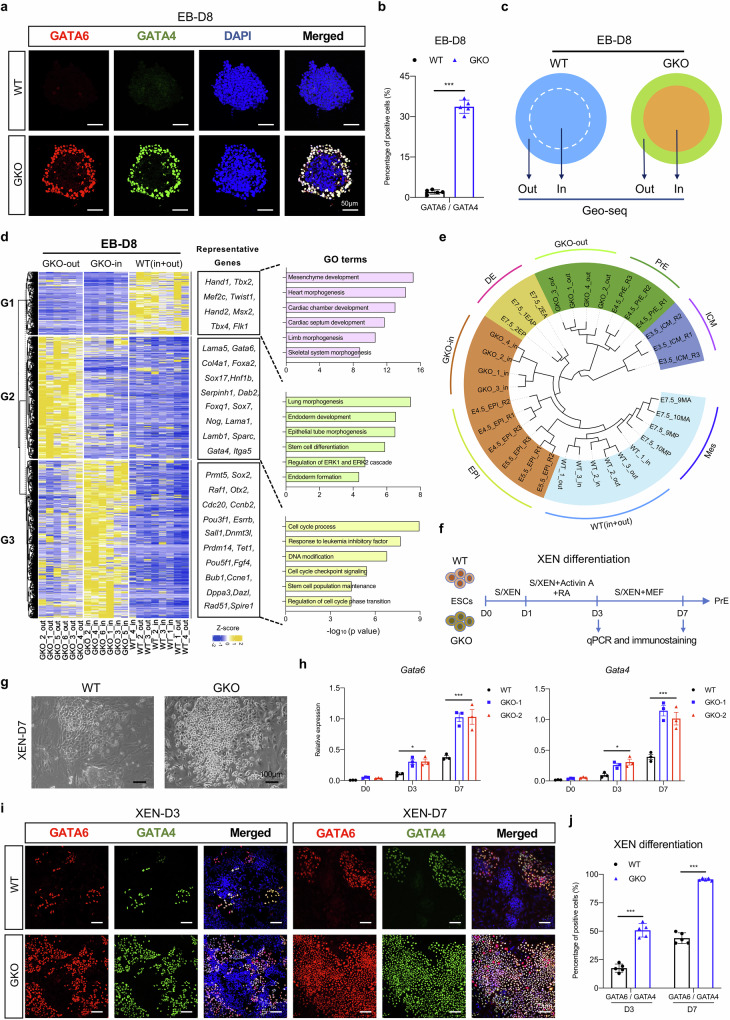
*Gm26793* knockout in mESCs boosts the responsiveness to PrE differentiation signals. **a**, **b** Immunofluorescence analyses showing the specific distribution of GATA6/GATA4-positive cells in the outer layer of GKO EBs collected at day 8. Quantification of fluorescent-positive cells is shown in **b** (*n* = 5). **c** Illustration showing the sample collection of outer and inner layers in WT and GKO EBs by Geo-seq for transcriptomic analysis. **d** DEG analyses identify the specific gene expression pattern for each dissected WT and GKO sample. Representative genes and GO terms of each DEG group are listed on the right panel. **e** The phylogenetic tree displaying the clustering of EB differentiation samples with mouse pre-implantation cell lineages as well as definitive endoderm and mesoderm lineages. The mouse pre-implantation, definitive endoderm and mesoderm transcriptomic data are collected from published datasets^63,70^. **f** The schematic diagram describing the differentiation protocol for XEN-directed differentiation from embryonic stem cells. **g** The morphologies of differentiated cells in bright field for both WT and GKO XEN cells at day 7. **h** Temporal expression dynamics of Gata6 and Gata4 during WT and GKO XEN differentiation. **i**, **j** Representative images showing the presence of GATA6/GATA4-positive cells in WT and GKO groups at day 3 and 7 during XEN differentiation. Quantification of the data is shown in **j**. All data are shown as means ± SEM. Student’s *t*-test analysis is used in **b** and **j**; Two-way ANOVA analysis with Tukey’s test is used in **h**; *P < 0.05, ***P < 0.001; *n* represents distinct EBs.

To characterize the differentiation phenotype of GKO cells, we analyzed the enrichment of endoderm-related markers in the differentiated EBs. As expected, we did not detect any expression of endoderm markers, such as GATA6 and SOX17, in the WT EBs (Fig. 2a, b; Supplementary Fig. S3a–d). Interestingly, in contrast to the uniform mesodermal cell distribution in WT EBs, EBs acquired in GKO groups exhibit a two-layer concentric circular structure where cells residing in the inner layer are densely structured, while cells residing in the outer layer are loosely organized and show a strong enrichment with endoderm protein signatures (Fig. 2a, b; Supplementary Fig. S3c, d). To further investigate the molecular features of the GKO EBs, we utilized Geo-seq^69^ to specifically profile the transcriptome of inner and outer layers of GKO EBs at D8 (Fig. 2c), and found that inner and outer cells of GKO group exhibit distinct transcriptomic patterns, unlike the indistinguishable cell composition in WT samples (Fig. 2d; Supplementary Fig. S3e). As revealed by the differentially expressed gene (DEG) analyses (Supplementary Table S3), we found the absence of mesodermal gene expression in both inner and outer layers of GKO EBs, which are highly expressed in WT EBs. Furthermore, the inner cell layer of GKO EBs expresses a higher level of pluripotency-related genes, such as *Prmt5*, *Sox2*, and *Pou5f1*. By contrast, cells residing in the outer layer of GKO EBs showed enrichment of endoderm-related genes, such as *Gata6*, *Foxa2*, and *Sox17* (Fig. 2d). These results suggested that GKO cells fail to initiate mesoderm differentiation, but tend to maintain a stem cell state in the inner layer of EBs, and are more likely to adopt an endoderm fate for cells in the outer layer, which are directly exposed to the signal stimulation.

To address the lineage features of the GKO cells, we incorporated a published embryogenesis in vivo dataset and built a hierarchy of clusters with differentiated EB samples (Fig. 2e). According to the hierarchical clustering results, we found that the inner cells of GKO EBs were clustered with epiblast samples around peri-implantation stages (E4.5_EPI and E5.5_EPI), when epiblast cells still maintain a naïve or formative pluripotent state^70–72^. Whereas, the outer layer of GKO EBs was closely linked with primitive endoderm samples (E4.5_PrE), but not definitive endoderm samples (E7.5_EA/P) (Fig. 2e). Consistently, we observed that, in contrast to the increased expression of pan-endoderm markers (Fig. 1h; Supplementary Fig. S3a), the expression of mesendodermal markers, *Eomes* and *Gsc*^73–76^, was severely affected (Supplementary Fig. S3f). Therefore, the increment of pan-endodermal markers’ expression, such as *Gata6* and *Sox17*, should be attributed to elevated primitive endoderm differentiation.

In view of the enhanced responsiveness to primitive endoderm commitment upon *Gm26793* knockout, we explored whether this preference is retained in the directed extraembryonic endoderm (XEN) differentiation system (Fig. 2f)^77,78^. Along with 7 days of differentiation, we found that nearly all GKO cells turned into highly refractile phase-bright XEN, a typical morphological characteristic of mature XEN cells (Fig. 2g). By contrast, only a subset of WT cells could achieve the epithelial-like XEN state, and the majority of WT cells remain in undifferentiated compact status. qPCR analysis showed the expression levels of primitive endoderm markers, such as *Gata6*, *Gata4*, *Sox17*, *Foxa2*, *Sox7*, and *Dab2*, were gradually up-regulated in both WT and GKO cells (Fig. 2h; Supplementary Fig. S3g). But, the increment of primitive endoderm markers in GKO cells was much faster than in WT cells. Similarly, immunostaining results also revealed that the protein signatures of primitive endoderm were markedly elevated in GKO cells (Fig. 2i, j; Supplementary Fig. S3h, i), which indicates that *Gm26793* knockout indeed boosts the differentiation of mESCs towards the primitive endoderm lineage.

### *Gm26793* null embryos exhibit developmental failure during early lineage segregation between epiblast and primitive endoderm in vivo

**Fig. 3:**
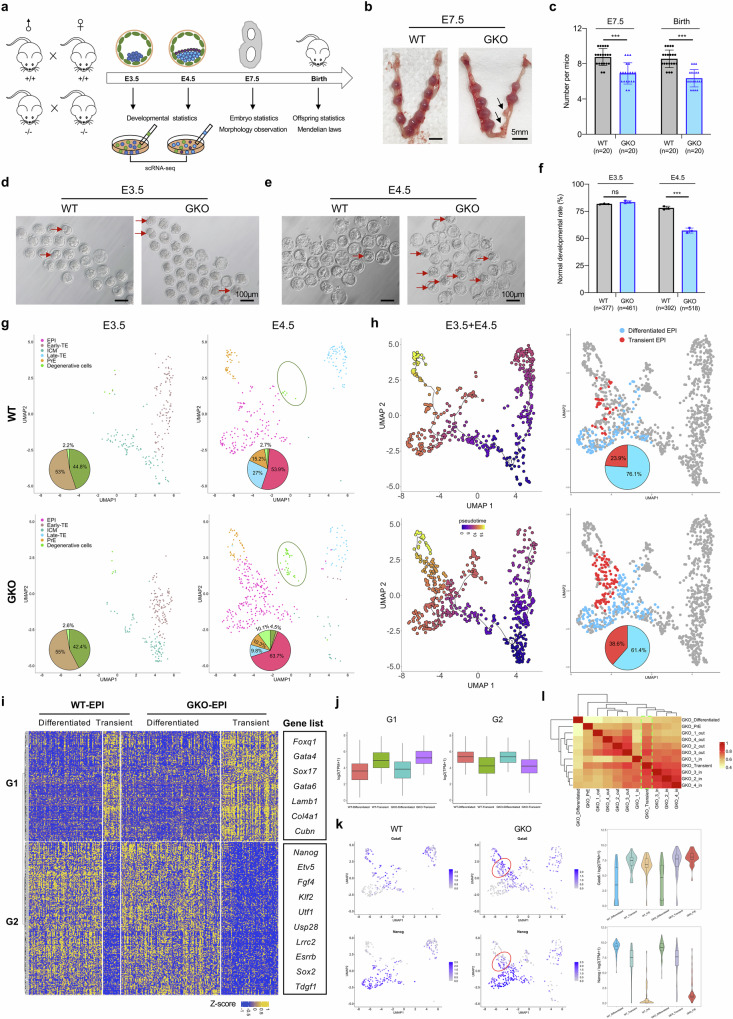
*Gm26793*-null embryos exhibit developmental abnormalities during the lineage segregation between epiblast and primitive endoderm. **a** Experimental overview of mouse breeding, embryo preparation and the functional assays conducted at the indicated developmental stages. **b** Representative images showing the resorbed decidua in GKO mouse at E7.5, which were highlighted by the arrows. **c** Quantification of the normal decidua collected at E7.5 and the acquired offspring number per litter. *N* represents the number of mating females. **d**, **e** Representative images showing the embryos collected at early (E3.5) (**d**) and late (E4.5) (**e**) blastocyst stage for both WT and GKO groups. Abnormal embryos were indicated by red arrows. **f** Quantification of the normal developmental rates at early and late blastocyst stage. *N* represents the total number of blastocysts collected. Data are summarized from three biologically independent experiments. **g** UMAP plots of scRNA-seq data from E3.5 and E4.5 blastocysts, respectively. The percentages of cell type composition are highlighted as a pie chart embedded in the related UMAP plot. Degenerative cells are circled by the oval. **h** Pseudotime trajectories of pre-implantation cell lineages inferred by Monocle 3 (left). Visualization of differentiated and transient epiblast cells in UMAP plots based on the trajectory branch towards primitive endoderm (right). The percentages of epiblast subtype cells are highlighted as a pie chart embedded in the related UMAP plot. **i** Heatmap showing DEG groups among different epiblast subtypes displayed in **h**. Representative genes are listed on the right panel. **j** Box plots showing average expression levels of differentiated and transient epiblast-specific genes in the indicated cell clusters. **k** Antagonistic distribution and expression level of primitive endoderm and epiblast marker genes among transient and differentiated epiblast cells. **l** Heatmap showing the transcriptomic correlation within in vitro EBs, differentiated and transient epiblast. All data are shown as means ± SEM. Student’s *t*-test analysis is used in **c**, **f**; ***P < 0.001.

To determine the roles of *Gm26793* during mouse embryogenesis, we generated *Gm26793* knockout mice by removing the same genomic region as GKO cells (Supplementary Fig. S4a). Generally, mice without *Gm26793* were viable. However, after summarizing the genotype of mice acquired from heterozygous parents, we found a non-negligible consistent loss of homozygous offspring (Supplementary Fig. S4b), which implied that a portion of homozygous GKO mice could be subjected to developmental failure at the embryonic stage. Following this, we internally crossed the knockout mice and collected the embryo samples at the pre-implantation (E3.5 and E4.5), post-implantation (E7.5), as well as postnatal stage (Fig. 3a), and then observed the statistical loss of viable individuals per litter at the postnatal stage, a decrease of normal gastrula as well as defective decidualization in the uterus at the early post-implantation stage (Fig. 3b, c; Supplementary Fig. S4c). Meanwhile, the expression of mesoderm markers, *T* and *Mesp1*, in E7.5 GKO with normal morphological embryos seemed to show no obvious distinctions compared to WT counterparts (Supplementary Fig. S4d). The comparable ratio of GKO embryo loss between post-implantation and postnatal stages illustrates that the in vivo function of *Gm26793* may act earlier than the gastrulation stage. Additionally, the lack of aberrant mesoderm developmental phenotype in GKO embryos indicates that the in vitro mesodermal differentiation defect (Fig. 1h; Supplementary Fig. S2h, i) may be a byproduct of resistance to exit pluripotency and enhanced primitive endoderm differentiation capacity in GKO cells.

To deeply delve into the potential function of *Gm26793* in the pre-implantation embryo, we collected early embryos at both E3.5 and E4.5 stages (Supplementary Table S4), in which the embryos start to form blastocyst consisting of three distinct lineages, trophoblast, epiblast, and primitive endoderm^79^, and found that the developmental rate of normal embryos between WT and GKO group seems to be equivalent at the E3.5 stage (Fig. 3d, f). However, once the embryos developed to the E4.5 stage, about 20% of GKO embryos exhibited blastocyst cavity formation defects (Fig. 3e, f). These results indicate that GKO embryos start to display developmental defects in the pre-implantation stage from E3.5 to E4.5.

Next, to systematically dissect the molecular and cellular changes of aberrant blastocysts caused by *Gm26793* knockout, we conducted single-cell RNA sequencing (scRNA-seq) of both WT and GKO embryos at the E3.5 and E4.5 stages by using SMART-seq2 sequencing (Fig. 3a)^80^. A total of 439 cells from WT embryos and 568 cells from GKO embryos were collected. Based on uniform manifold approximation and projection analyses (UMAP) and marker gene expression (Supplementary Table S5), five known cell clusters were identified in the embryo samples, and annotated as epiblast, inner cell mass, early trophectoderm, late trophectoderm and primitive endoderm, respectively (Supplementary Fig. S4e, f, h). Notably, we found that one distinct cell group showed degenerative features, which manifests as a high level of apoptotic-related mitochondrial gene expression and majorly harbors cells in GKO embryos at the E4.5 stage (Fig. 3g; Supplementary Fig. S4e–g). Next, to capture the potential developmental phenotypes of the GKO embryo, we reconstructed the pseudotime lineages for cell types in both WT and GKO embryos using Monocle 3^81^. Following the trajectory of epiblast and primitive endoderm lineage segregation from inner cell mass, we determined two states of epiblast cells, transient and differentiated states, in both WT and GKO embryos (Fig. 3h). Statistic analysis identified that a greater proportion of GKO epiblast cells (38.6%) than WT epiblast cells (23.9%) were in the transient state, which expressed higher levels of primitive endodermal genes (G1) but lower levels of pluripotent genes (G2) than epiblast in the differentiated state (Fig. 3h–k; Supplementary Table S6). By integration with transcriptome data from EBs (Fig. 2c), we found that GKO_Transient cells also displayed higher correlation with both the inner and outer layer cells from GKO EBs than GKO_Differentiated cells (Fig. 3l; Supplementary Fig. S4j). As is known, the lineage segregation of inner cell mass cells into epiblast and primitive endoderm relies on an intricate balance between pluripotent genes (such as *Nanog*) and primitive endoderm regulators (such as *Gata6*)^82–84^. The disorganized expression of epiblast genes or primitive endoderm genes in transient state epiblast cells caused by *Gm26793* knockout, similar to the in vitro stem cell differentiation defects, can disrupt the proper lineage segregation process and further induce defects of blastocyst development in vivo.

### *Gm26793*-mediated regulation is independent of the transcript and local transcriptional activity

**Fig. 4:**
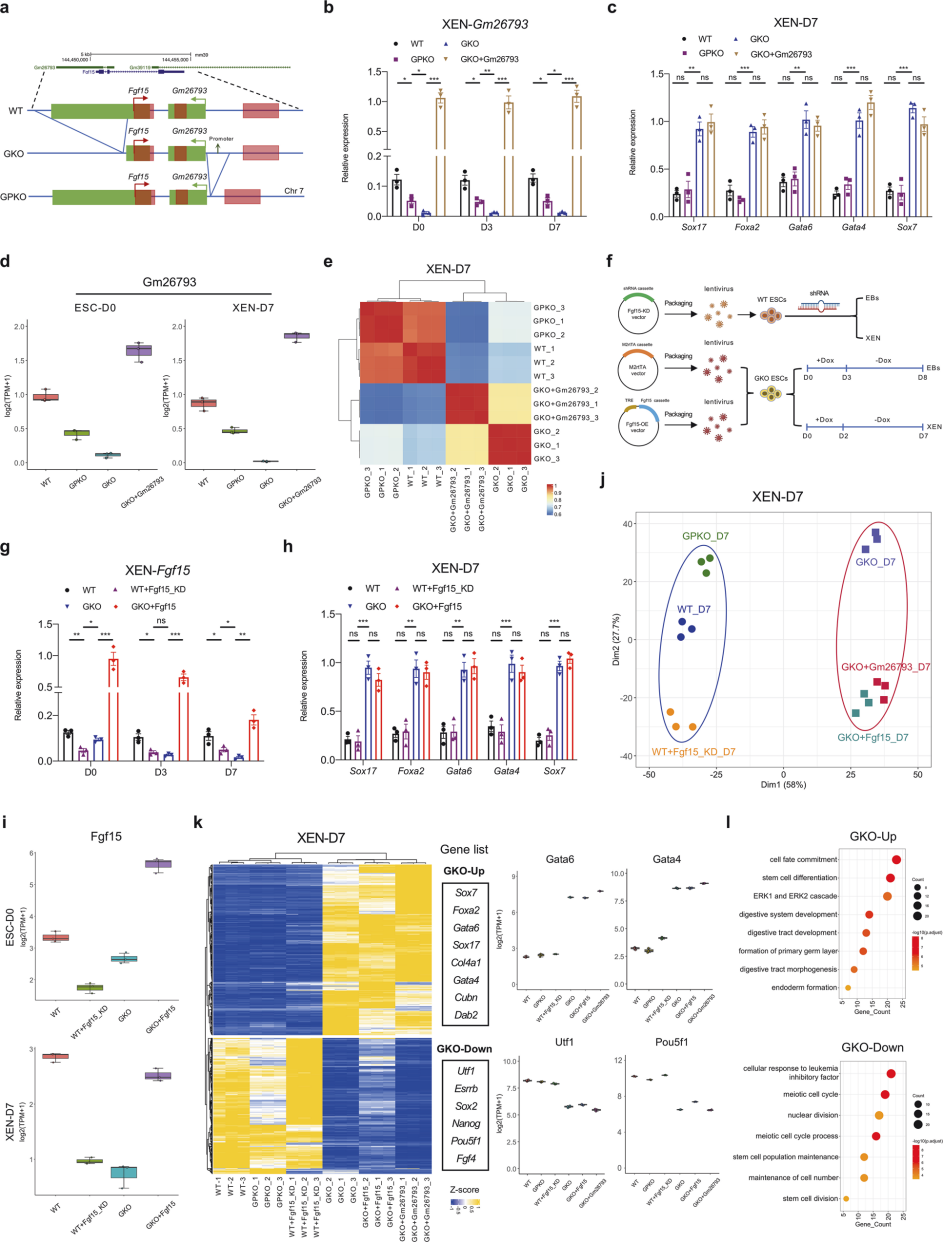
*Gm26793*-mediated regulation is independent of the transcript and local transcriptional activity. **a** The schematic diagram of the genomic information around *Gm26793* locus and the related promoter knockout strategy. **b** Time-series expression of *Gm26793* in GPKO and GKO + Gm26793 cells (*Gm26793* overexpression in GKO cells) during XEN differentiation. **c** qPCR analyses of primitive endoderm-related genes in the indicated cell groups on XEN-D7. **d** Quantification of *Gm26793* transcripts by RNA-seq analysis. **e** Transcriptomic correlation of XEN-D7 differentiation samples upon *Gm26793* transcript downregulation or overexpression. **f** The workflow for knocking down and overexpressing *Fgf15* during differentiation by lentivirus infection. **g** Bar plots showing the expression dynamics of *Fgf15* in the indicated cell groups during XEN differentiation. WT + Fgf15_KD represents knockdown of *Fgf15* in WT mESCs. GKO + Fgf15 represents conditional overexpression of *Fgf15* in GKO mESCs. **h** qPCR analyses of primitive endoderm markers among respective cell types on XEN-D7. **i** Quantification of *Fgf15* transcripts by RNA-seq analysis. **j** PCA analyses of XEN-D7 differentiation samples showing the transcriptome similarities of differentiated cells following the treatment with decrease and increase in *Gm26793* and *Fgf15* expression. **k**, **l** Heatmap showing the DEGs between the indicated cell groups. The expression changes of representative up- and down-regulated genes are listed on the right panel. Top GO terms of up- and down-regulated genes are shown in **l**. All qPCR data are shown as means ± SEM. Two-way ANOVA analysis with Tukey’s test is used in **b**, **g**; One-way ANOVA analysis with Tukey’s test is used in **c**, **h**; *P < 0.05, **P < 0.01, ***P < 0.001.

To examine the roles of *Gm26793* transcriptional elongation, RNA molecules or its genomic locus in cell fate determination, we generated cells by deleting the promoter region upstream of transcriptional start site of *Gm26793* (GPKO cells) (Fig. 4a; Supplementary Fig. S5a, b), or reintroduced full-length *Gm26793* transcript into the GKO cells through lentivirus infection (GKO + Gm26793). Of note, the disruption of local transcription events by knocking out the promoter, which leads to a great reduction of *Gm26793* transcript (Fig. 4b; Supplementary Fig. S5c), has limited effects on the differentiation towards mesoderm or primitive endoderm fate (Fig. 4c; Supplementary Fig. S5d, e). Moreover, even though overexpression of *Gm26793* could sufficiently restore the expression of *Gm26793* in GKO cells during both EB and XEN differentiation (Fig. 4b; Supplementary Fig. S5c), neither mesoderm markers nor primitive endoderm markers could be rescued (Fig. 4c; Supplementary Fig. S5d, e). Systematic analyses confirmed that the GPKO cells retain a highly correlated transcriptomic architecture of the WT control, while the GKO + Gm26793 still maintains an aberrant gene expression pattern like the GKO cells (Fig. 4d, e).

It has been reported that the local transcriptional activity could affect the expression of both lncRNA and its nearby protein-coding gene^85,86^. In this study, *Gm26793* is located at the divergent direction of the *Fgf15* locus with partial genomic overlap (Fig. 4a), and the knockout of *Gm26793* indeed leads to the down-regulation of *Fgf15* expression during both EB and XEN differentiation (Supplementary Fig. S5f). To explore the role of *Fgf15*, we knocked down *Fgf15* in WT mESCs by using shRNA (WT + Fgf15_KD) or conditionally over-expressed *Fgf15* in GKO cells (GKO + Fgf15), and then performed stem cell differentiation assay (Fig. 4f). Examination of maker gene expression indicated that neither significant down-regulation of *Fgf15* expression nor inducible over-expression of *Fgf15* at the early stage of differentiation affects the mesendoderm and primitive endoderm-related gene expression in comparison with the control group (Fig. 4g, h; Supplementary Fig. S5f, g). Besides, we also profiled the transcriptome of these cells during XEN differentiation, which also confirmed the successful modulation of *Fgf15* expression level in relevant cells (Fig. 4i). Integrative analyses with the transcriptome data for WT, GKO, GPKO, WT + Fgf15_KD, GKO + Gm26793 and GKO + Fgf15 cells demonstrated that the modulation of *Gm26793* or *Fgf15* expression level has no impact on the global transcriptome pattern (Fig. 4j; Supplementary Fig. S5h). Specifically, genes up-regulated in the GKO cells (GKO-Up), which were related to endoderm formation, were also highly expressed in the GKO + Fgf15 and GKO + Gm26793 cells. Concurrently, genes down-regulated in the GKO cells, which are involved with stem cell maintenance, failed to be down-regulated in the GPKO and WT + Fgf15_KD cells (Fig. 4k, l; Supplementary Table S7). Collectively, these results support that the transcriptional activity and transcripts of *Gm26793*, as well as adjacent coding gene-*Fgf15*, are dispensable for primitive endoderm differentiation.

### *Gm26793* regulates stem cell differentiation through direct inter-chromosomal interaction with *Cubn* locus

As reflected by the transcriptome data, we found that the knockout of *Gm26793* leads to severe developmental abnormalities, manifesting as the aberrant up-regulation of XEN-related genes (Figs. 1h, 2d, 3i, 4k). Detailed exploration of the transcriptome data revealed that the aberrant transcriptome starts to emerge in the form of the transient epiblast state in vivo (Fig. 3h) or at the stage of D0 ESCs in vitro (Supplementary Fig. S6a, b), when the expression of *Gm26793* is restricted (Fig. 1e; Supplementary Fig. S4i). Following this, we want to test whether the genomic locus of *Gm26793* could contribute to the differentiation defects, especially through remote chromatin interactions. To this end, we performed circular chromosome conformation capture sequencing (4C-seq) using the *Gm26793* knockout region as a bait to query genome-wide chromatin interactions. Among 1919 interacting targets, 1318 nearest neighboring genes (Supplementary Table S8) were finally assigned in three replicates of WT mESCs. Besides the relatively higher enrichment of intra-chromosomal peaks, we also detect the pervasive existence of interacting chromatin hits at the neighboring chromosomes (Supplementary Fig. S6c). These results denote that the genomic locus of *Gm26793* may act as a chromatin interaction hub by forming both intra- and inter-chromosomal interactions.

**Fig. 5:**
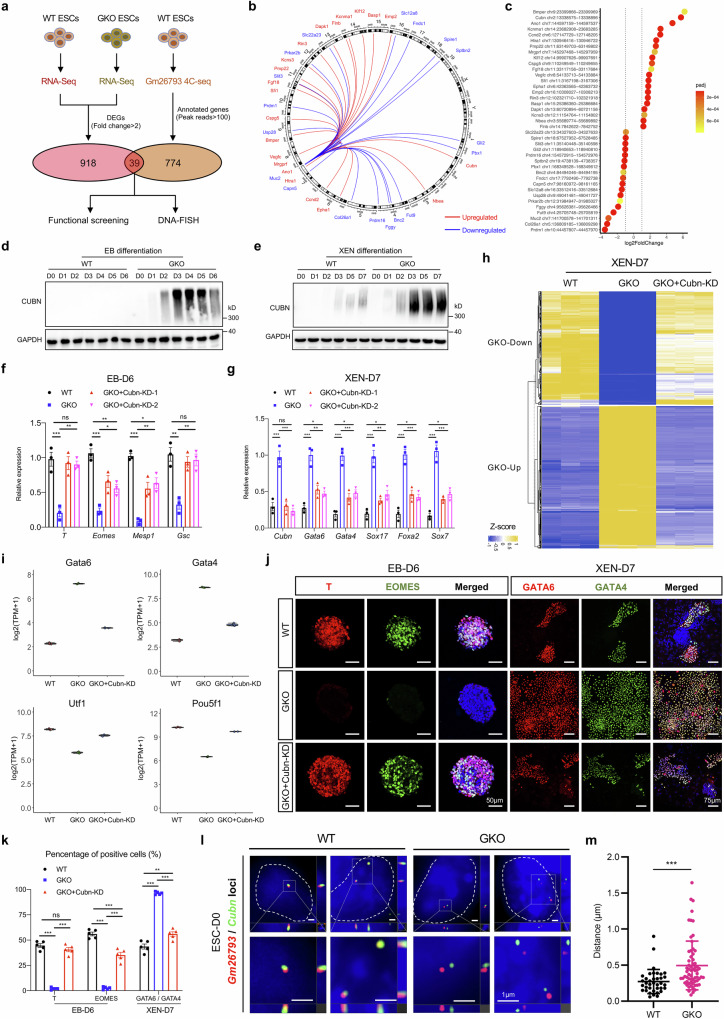
The genomic locus of *Gm26793* forms inter-chromosomal interaction with *Cubn.* **a** Venn diagram showing the workflow for the screening of potential *Gm26793* targets by the integrative analyses of RNA-seq and 4C-seq data. **b** Circos plot showing inter- and intra-chromosomal connectivity associated with the genomic region of *Gm26793*. Line colors represent up- (red) and down-regulation (blue) of annotated genes. **c** Dot plot summarizing genes with both significant *Gm26793*-interacting hits revealed by 4C-seq and obvious expression changes between WT and GKO cells based on transcriptomic data. Genes are ranked by the fold change of expression. **d**, **e** Western blotting results reporting the aberrant activation of CUBN in GKO cells during EB (**d**) and XEN (**e**) differentiation. **f**, **g** The expression recovery of mesoderm-associated genes on day 6 of EB differentiation (**f**) and PrE-related genes on day 7 of XEN differentiation (**g**) upon *Cubn* knockdown in GKO cells. **h**, **i** Heatmap showing the transcriptomic recovery of GKO samples on XEN-D7 upon *Cubn* knockdown. Expression recovery of representative up- and down-regulated genes are listed in **i**. **j**, **k** Representative images describing the rescue of protein distribution during EB (left) and XEN (right) differentiation. Scale bars, 50 μm and 75 μm. Quantification of the data is shown in **k**. **l**, **m** dCas9-mediated living cell imaging showing the spatial separation of the *Gm26793* (Red probe) and *Cubn* (Green probe) loci upon GKO region deletion in mESCs. The increase in 3D-distance between *Gm26793* and *Cubn* loci is presented in **m** as mean ± SD (WT, *n* = 37 cells; GKO, *n* = 65 cells). One-way ANOVA analysis with Tukey’s test is used in **f**, **g**, **k** and data are shown as means ± SEM; Student’s *t*-test analysis is used in **m**; *P < 0.05, **P < 0.01, ***P < 0.001.

To refine the specific functional targets for the *Gm26793* locus, we combined the transcriptome data (Supplementary Table S9) of WT and GKO mESCs with 4C-seq data, and screened out 39 gene candidates with significant gene expression changes (21 genes were up-regulated, 18 genes were down-regulated) triggered by *Gm26793* knockout (Fig. 5a–c). RT-qPCR analyses further corroborated that 18 out of 21 up-regulated genes and 13 out of 18 down-regulated genes were truly altered in GKO cells (Supplementary Fig. S6d,e). Furthermore, by referencing the published datasets^87–92^, we eventually focused on eight genes with potential roles in stem cell differentiation, including five upregulated genes (*Cubn, Ano1*, *Htra1*, *Sfi1*, and *Flnb*) and three down-regulated genes (*Slc12a8*, *Usp28*, *Fut9*). Then, we established cell lines with knocking down the up-regulated genes or over-expressing the down-regulated genes in GKO cells, respectively (Supplementary Fig. S6f). The restoration of 7 selected genes (*Ano1*, *Htra1*, *Sfi1*, *Flnb*, *Slc12a8*, *Usp28*, *Fut9*) failed to recover the expected cell lineage transition during EBs and XEN differentiation (Supplementary Fig. S6g, h). On the contrary, for the gene of *Cubn*, which was up-regulated in GKO cells during differentiation and linked by inter-chromosomal interaction with *Gm26793* locus (Fig. 5b–e; Supplementary Fig. S7a–c, e), we found that knockdown of *Cubn* expression level in GKO cells could partially rescue the differentiation phenotype (Fig. 5f–k; Supplementary Fig. S7e–j) and was sufficient to restore the molecular abnormalities caused by *Gm26793* fragment deletion through transcriptomic profiling (Fig. 5h, i; Supplementary Fig. S7d). These results indicate that *Cubn* could be the direct interaction target of *Gm26793*, and the elevated expression of *Cubn* should be responsible for the raised primitive endoderm differentiation potential in GKO cells. Subsequently, to unambiguously verify the inter-chromosomal association between *Gm26793* and *Cubn* loci, we took advantage of the CRISPR-dCas9-assisted live imaging system^93^ and designed specific crRNA probes targeting the genomic locations of *Gm26793* and *Cubn*, to visualize the spatial distribution of each locus in the WT and the GKO living mESCs. As predicted, the genomic loci of *Gm26793* and *Cubn* were spatially co-localized in the WT nuclei, whereas the genomic loci became separated in the GKO cells (Fig. 5l, m).

Taken together, these data unveil that *Gm26793* in chromosome 7 forms an inter-chromosomal interaction with *Cubn* in chromosome 2, and the specific deletion of *Gm26793* locus can release this contact and enhance the expression level of *Cubn* during the stem cell differentiation process, which supports the notion that the inter-chromosomal organization between *Gm26793* and *Cubn* loci can behave as a molecular lock to restrict the expression of *Cubn* in WT cells.

### The pervasive remodeling of the epigenomic landscape in GKO cells could be restored by silencing *Cubn* expression

**Fig. 6:**
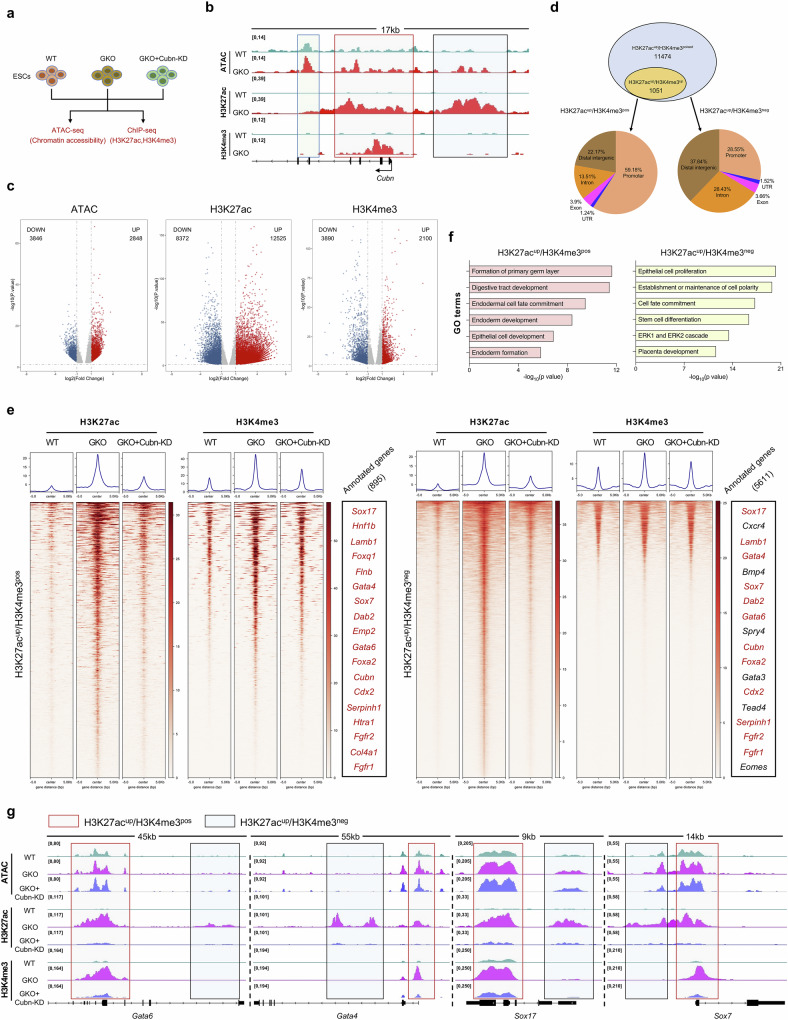
*Gm26793* knockout leads to pervasive epigenomic remodeling, which can be rescued by knocking down *Cubn* expression. **a** Schematic diagram of epigenomic profiling among related mESCs. **b** Genome browser snapshot showing the increased chromatin accessibility, H3K27ac and H3K4me3 enrichment around the *Cubn* locus in GKO mESCs. **c** Volcano plot determining the differential distribution of chromatin accessibility (left), H3K27ac (middle), and H3K4me3 (right) between WT and GKO cells. Enhanced signals in GKO cells are highlighted in red, and decreased signals in GKO cells are highlighted in blue. **d** Venn diagram showing genomic regions with increased H3K27ac in GKO cells, which are analyzed and divided into H3K27ac^up^/H3K4me3^pos^ and H3K27ac^up^/H3K4me3^neg^ elements based on the existence of H3K4me3 modification. Genomic annotations of divided regions are presented as pie chart. **e** Heatmaps showing the signal enrichment of H3K27ac and H3K4me3 around selected genomic regions (H3K27ac^up^/H3K4me3^pos^: left; H3K27ac^up^/H3K4me3^neg^: right). Representative annotated genes for the selected regions are also shown. Identical genes are labeled in red. **f** GO term enrichment for the genes in H3K27ac^up^/H3K4me3^pos^ group (left) and H3K27ac^up^/H3K4me3^neg^ group (right). **g** Representative genome browser snapshot showing efficient recovery of epigenetic distribution in GKO + Cubn-KD cells. H3K27ac^up^/H3K4me3^pos^ regions are highlighted in light red rectangles, H3K27ac^up^/H3K4me3^neg^ regions are highlighted in light blue rectangles.

To understand the molecular basis of the facilitated primitive endoderm differentiation potential decribed above, we analyzed the global epigenomic pattern in both WT and GKO cells by profiling the genomic distribution of chromatin-accessible regions, active histone marker H3K27ac, as well as promoter-related histone marker H3K4me3 (Fig. 6a) and observed a consistent elevation of chromatin accessibility, H3K4me3 and H3K27ac enrichment around *Cubn* locus (Fig. 6b). By systematic comparison between WT and GKO cells, we found that the epigenomic pattern including chromatin accessibility and histone modification distributions were pervasively altered, in which the global distribution of H3K27ac exhibits the most dramatic changes in GKO cells (Fig. 6c; Supplementary Fig. S8a and Table S10). Considering that the enrichment of H3K27ac has been used as an epigenetic marker to demarcate the primed and activated chromatin states of regulatory elements, the dramatic alteration of H3K27ac in GKO cells indicates that widespread chromatin state transition may occur in GKO cells, which may be related to the enhanced responsiveness to primitive endoderm. Following this, we focused on analyzing chromatin regions with up-regulated H3K27ac in GKO cells and further clustering these regions based on the existence of promoter-related histone marker H3K4me3 (Fig. 6d). By dividing these regions into newly derived H3K27ac^up^/H3K4me3^pos^ elements (H3K4me3 and H3K27ac double positive), and newly derived H3K27ac^up^/H3K4me3^neg^ elements (H3K27ac positive only) in GKO cells, we found that these newly derived regulatory elements were mostly enriched around endoderm-related genes, such as *Gata6*, *Gata4*, *Sox17* and *Sox7* (Fig. 6e–g; Supplementary Fig. S8b, c and Table S11). Besides, we found that the relative enrichment of H3K27ac at both H3K27ac^up^/H3K4me3^pos^ and H3K27ac^up^/H3K4me3^neg^ regions in GKO cells can be largely erased by knocking down *Cubn* expression, and return to a comparable level with WT cells (Fig. 6e, g; Supplementary Fig. S8d). Thus, the genetic knockout of *Gm26793* leads to pervasive remodeling of the epigenomic landscape, especially for global re-distribution of active marker H3K27ac around endodermal genes, and the remodeling of active H3K27ac modification can be largely revised by knocking down *Cubn* expression.

### CTCF orchestrates the inter-chromosomal association between *Gm26793* and *Cubn* loci

**Fig. 7:**
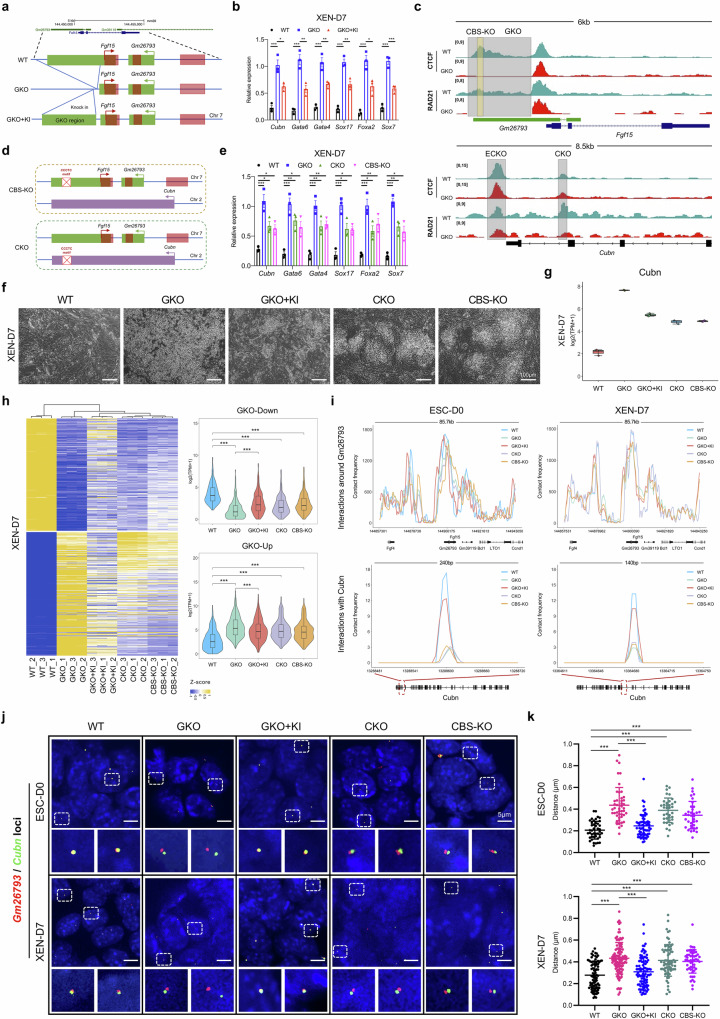
CTCF orchestrates the inter-chromosomal association between *Gm26793* and *Cubn* loci. **a** Schematic diagram showing the strategy for GKO fragment knockin. **b** Bar plot showing the partial rescue of primitive endoderm markers upon GKO region knockin on XEN-D7. **c** Genome browser view demonstrating the binding of CTCF and RAD21 at *Gm26793* and *Cubn* loci in WT and GKO mESCs. Grey boxes represent the co-binding sites of CTCF and RAD21, which were subjected to genomic knockout in WT cells and following experiments. The light yellow box represents the CCCTC motif within GKO region. **d** Schematic illustration depicting the specific knockout of CTCF-binding site within the *Gm26793* (CBS-KO) and *Cubn* (CKO) loci. **e** Relative expression changes of primitive endoderm-related genes on XEN-D7 upon CTCF binding site knockout. **f** Morphologies of differentiated cells at bright field in the indicated cell groups. **g** Quantification of *Cubn* expression by RNA-seq analysis on XEN-D7. **h** Heatmap showing the DEGs upon CTCF-binding site knockout or knockin on XEN-D7. The average expression levels of up- and down-regulated genes are listed on the right panel. **i** Quantification of the contact frequency with neighboring regions and *Cubn* locus by using *Gm26793* as a viewpoint on ESC-D0 and XEN-D7 in the indicated cell groups. **j** Representative images recording the spatial distribution of the *Gm26793* and *Cubn* loci in the indicated cells on ESC-D0 and XEN-D7. Scale bars, 5 μm. (ESC-D0: WT, *n* = 43 cells; GKO, *n* = 46 cells; GKO + KI, *n* = 62 cells; CKO, *n* = 3 6 cells; CBS-KO, *n* = 36 cells. XEN-D7: WT, *n* = 84 cells; GKO, *n* = 105 cells; GKO + KI, *n* = 95 cells; CKO, *n* = 7 0 cells; CBS-KO, *n* = 52 cells). **k** Scatter plots showing the statistical changes of 3D-distance between the *Gm26793* and *Cubn* loci in the indicated cell groups on ESC-D0 (top) and XEN-D7 (bottom). qPCR data are shown as means ± SEM. 3D-distance statistics are shown as means ± SD. One-way ANOVA analysis with Tukey’s test is used in **b**, **e**, **h**, **k**; *P < 0.05, **P < 0.01, ***P < 0.001.

Next, to rigorously determine the necessity of the *Gm26793* sequence on *Cubn* suppression, we re-introduced the GKO region (~1.5 kb) approximately 380 bp downstream of the original deletion site in the GKO mESCs (named as GKO + KI) (Fig. 7a; Supplementary Fig. S9a, b). Intriguingly, the reintroduction of *Gm26793* fragment significantly down-regulated the expression of *Cubn* and other primitive endoderm markers (Fig. 7b). Previously, ChIA-PET analyses showed that CTCF and cohesin extensively participate in the inter- and intra-chromosomal interactions and configure the genome into distinct domains that possess unique epigenetic states^26,44,94^. To further delineate the mechanism of the inter-chromosomal molecular lock within *Gm26793*-*Cubn* loci, we performed CTCF and RAD21 ChIP-seq in both WT and GKO mESCs. Globally, the genomic distributions of CTCF and RAD21 within peak center ± 5 kb in both cells remain largely unchanged (Supplementary Fig. S9c and Table S12). As to *Gm26793* and *Cubn* loci, we found both CTCF and RAD21 are co-bound at the *Gm26793* knockout region, as well as around the *Cubn* locus (Fig. 7c). Then, we knocked down the expression of *Ctcf* and *Rad21* in WT mESC, respectively, and found that the decrement of *Ctcf* expression could obviously upregulate *Cubn* expression, whereas *Rad21* downregulation did not affect *Cubn* expression (Supplementary Fig. S9d, e). These results infer that the inter-chromosomal association between *Gm26793* and *Cubn* loci may be fixed by CTCF, just like a molecular lock, at the highlighted regions (Fig. 7c).

To assess the roles of CTCF enrichment, we refined the genome editing strategy by precisely depleting the CTCF-binding sites within *Gm26793* and *Cubn* non-coding region (named as ECKO, CKO, and CBS-KO, respectively) (Fig. 7c, d; Supplementary Fig. S9f–l), and performed XEN differentiation assay afterwards. For the ECKO cells (knockout of CCCTC motif adjacent to the *Cubn* gene region), we found that the expression of *Cubn*, as well as the primitive endoderm markers, has not been changed (Supplementary Fig. S9h). By contrast, the expression of *Cubn* was evidently up-regulated in CKO (knockout of CCCTC motif within the *Cubn* intron region) and CBS-KO (knockout of CCCTC motif within the *Gm26793* gene region) cells, albeit with a less extent than GKO cells (Fig. 7e). Systematic examination of the morphological features during XEN differentiation confirmed that the CKO and CBS-KO cells showed the appearance of boosted primitive endoderm differentiation preference, which recapitulated the differentiation phenotype of GKO cells, while the presence of refractile phase-bright XEN cells was decreased in the GKO + KI cells (Fig. 7e, f; Supplementary Fig. S10a, b). Then, through transcriptomic analyses, we found that both the CKO and CBS-KO cells could phenocopy the molecular defects observed in the GKO cells, whereas GKO fragment re-insertion (GKO + KI) partially restored the gene expression abnormalities (Fig. 7g, h; Supplementary Fig. S10c). Moreover, epigenomic profiling revealed that the increment of active epigenomic features at endoderm-related genes present in GKO cells, including *Cubn*, *Gata6*, *Gata4*, and *Sox17*, was also reproduced in CKO cells (Supplementary Figs. S8d, S10d, e). Pioneer exploration using a published ChIP-seq dataset^95^ reported that the repressive histone marker H3K27me3, as well as key components of the PRC2 complex, were deposited at the *Gm26793* locus but not the CKO locus in WT ESCs, when *Cubn* is not expressed at this stage (Supplementary Fig. S10f). Next, we checked the distribution of H3K27me3 around the *Gm26793* locus in the cell lines with and without this inter-chromosomal interaction, and found that deletion of the CTCF-binding site at each end (GKO, CKO, CBS-KO) could significantly reduce the abundance of H3K27me3. However, re-introduction of *Gm26793* loci in GKO cells failed to recover the lost H3K27me3 modification (Supplementary Fig. S10f). Together, these results support that the *Gm26793*-*Cubn* inter-chromosomal interaction bridged by CTCF may be coupled with PRC2 complex, and then creates a repressive chromatin microenvironment to safeguard *Cubn* expression, thus ensuring proper primitive endoderm differentiation.

Finally, to faithfully reflect the indispensability of CTCF binding in orchestrating the *Gm26793*–*Cubn* inter-chromosomal association, we applied 4C-seq and Tn5-based DNA-FISH imaging to visualize the contact frequency changes and spatial localization of both loci in all acquired cell lines, and discovered that the interaction frequencies severely decreased upon CTCF-binding site knockout (CKO and CBS-KO cells) in comparion with WT cells, and the re-introduction of *Gm26793* fragment in the GKO cells (GKO + KI cells) can significantly enhance the inter-chromosomal interaction on both D0 and D7 (Fig. 7i). Similarly, DNA-FISH analyses demonstrated that the originally tight co-localized *Gm26793* and *Cubn* loci were separated apart in both CKO and CBS-KO cells, manifesting as increasing spatial distance between the detected FISH signals compared to the WT control, whereas the re-incorporation of GKO region into GKO cells was sufficient to re-establish the trans-interaction between *Gm26793* and *Cubn* loci (Fig. 7j, k; Supplementary Fig. S10g). These results highlight the crucial roles of both GKO and CKO loci in forming the inter-chromosomal interaction, and the binding of CTCF is directly responsible for establishing the inter-chromosomal contact.

## Discussion

**Fig. 8:**
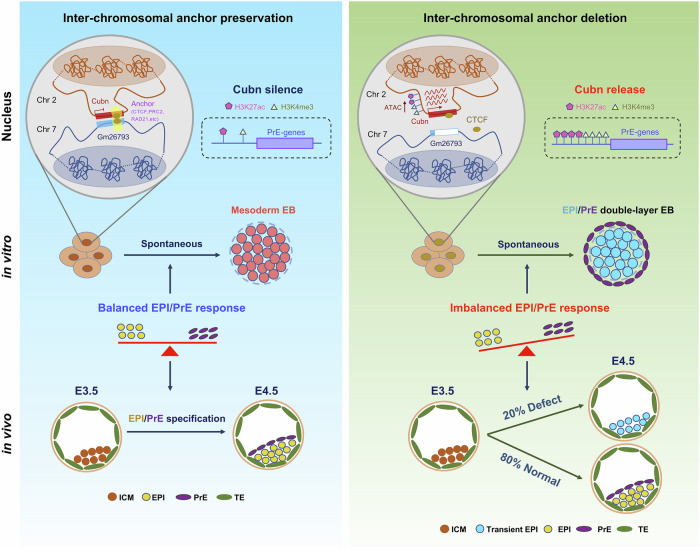
The inter-chromosomal anchor model. The schematic highlights the function and mechanism of *Gm26793*-*Cubn* inter-chromosomal interaction in primitive endoderm lineage commitment.

In this study, we identify a germ layer-specific lncRNA gene, *Gm26793*, through comprehensive analyses of the spatial transcriptome atlas of the mouse gastrula (Fig. 1a). To investigate its functional significance, we established GKO mESCs and mouse models by genetically knocking out *Gm26793*. During in vitro spontaneous differentiation, we find a significant impairment in the expression of mesendodermal and mesodermal genes (Fig. 1f; Supplementary Fig. S3f), along with abnormal up-regulation of genes related to primitive endoderm development (Fig. 2a; Supplementary Fig. S3a). Meanwhile, about 20% of embryos lacking the *Gm26793* locus also exhibit developmental arrest during the lineage segregation between epiblast and primitive endoderm fate, which further results in decidualization abnormalities at the post-implantation stage, as well as a decrease in normal individual per litter during the mouse life cycle (Fig. 3b–f). Towards an in-depth understanding of the molecular basis for *Gm26793* function, we modulated the expression of *Gm26793* and the neighboring *Fgf15* gene in WT and GKO cells, respectively. However, no functional rescue could be observed, indicating that the function of *Gm26793* is independent of its own transcript and proximal gene (Fig. 4). As revealed by 4C-seq and live cell FISH, we unravel that the genomic locus of *Gm26793* in chromosome 7 can directly interact with *Cubn* (Fig. 5b, l), a primitive endoderm regulator located in adjacent chromosome 2^92^. Molecularly, this specific inter-chromosomal interaction is bridged by CTCF (Fig. 7c), and specific depletion of the CTCF-binding site residing in both ends of the inter-chromosomal interaction, respectively, breaks up the inter-chromosomal interaction, releases the restriction of *Cubn* expression, remodels the epigenomic landscape in mESCs, and finally forms a primitive endoderm signal-responsiveness state (Figs. 6, 7). Notably, these effects can be restored by knocking down of *Cubn* (Figs. 5f–k, 6e, g) or re-insertion of the GKO fragment (Fig. 7a, b, f–k). Taken together, we propose an “inter-chromosomal silencer” model to elucidate the necessity and sufficiency of chromatin architectural protein CTCF-mediated chromatin interaction between *Gm26793* and *Cubn* loci in controlling *Cubn* expression and directing proper stem cell differentiation and mouse embryo development (Fig. 8).

We determined that *Gm26793* functions as a crucial regulator for both stem cell differentiation and early embryogenesis. LncRNAs have been revealed to fine-tune biological processes and regulate the spatiotemporal expression of pleiotropic developmental loci instead of being master regulators or switches of development^96^. A considerable portion of lncRNAs have been reported to be dispensable, albeit with context-dependent function during mammalian development^97^. Thus, a comprehensive evaluation of the functional relevance of lncRNA genes warrants dual integration of in vitro and in vivo systems. Here, we generated both embryonic stem cells and a mouse model with *Gm26793* locus removal. As revealed in the in vitro spontaneous differentiation system, the GKO EBs, which are supposed to express mesodermal genes, exhibit two-layer structures with primitive endodermal genes enriched at the outer layer and pluripotent genes enriched at the inner layer (Fig. 2d). Hence, for the in vitro system, *Gm26793* knockout either leads to cell arrest at the pluripotent state, or those that successfully exit the pluripotent state, are prone to differentiate into primitive endoderm fate. Consistently, in the in vivo mouse model, we detect a substantial incidence of blastocyst formation defects in GKO embryos (Fig. 3e, f). As revealed by scRNA-seq results of relevant embryos, we find that *Gm26793* was not expressed in the normal blastocysts (Supplementary Fig. S4i) and observe the over-representation of epiblast cells in degenerative or transient states for GKO embryos (Fig. 3g, h), which underscores the reason for the higher frequency of developmental failure upon *Gm26793* deficiency. The formation of a normal blastocyst relies on the timely separation and balanced expression of lineage-specific genes between epiblast and primitive endoderm cells^98–100^. The elevated proportion of transient epiblast cells, which act as the in vivo counterpart for the in vitro GKO EBs with strong co-expression of pluripotent and primitive endodermal genes (Fig. 3l), disrupts the cellular homeostasis and sequential gene expression programs for the lineage segregation during early embryogenesis. Given that a considerable proportion of GKO mice remain viable with normal morphology, some alternative compensatory mechanisms may exist in vivo to restore the developmental defects caused by *Gm26793* knockout.

We report the existence of functional inter-chromosomal interaction involving an lncRNA gene. As a newly defined regulatory dimension, various lncRNAs have been identified and reported to be involved in the modulation of chromatin function, alteration of cytoplasmic mRNAs’ stability and translation, as well as interference with signaling pathways^101,102^. In many cases, the abundance of lncRNA transcripts and the adjacent genes have been implicated in their biological functions. However, in this study, the modulation of *Gm26793* or the neighboring *Fgf15* gene expression fails to rescue the developmental defects caused by *Gm26793* knockout (Fig. 4). Interestingly, the refined genome manipulation by just knocking out the CTCF-binding site in WT cells, which does not impact *Fgf15* expression, faithfully recapitulates the morphological and molecular phenotypes detected in the GKO cells, while re-inserting the deleting *Gm26793* fragment in the GKO cells can partially rescue the phenotypes (Fig. 7). Taken together, these results demonstrate that the reported phenotype could not be attributed to the potential functional overlap among the *Gm26793* locus, *Fgf15* regulatory regions, and *Fgf15* gene. Even though no obvious similarities could be detected between *Gm26793*-knockout and *Fgf15*-interrupting cells in vitro, we propose that a systematic comparison using in vivo GKO and *Fgf15* mutant embryos will further clarify the relevance between the *Gm26793* locus and *Fgf15* regulation. Investigation of the corresponding genomic locus by capturing potential interacting chromatin regions reveals the existence of direct inter-chromosomal interaction between *Gm26793* and *Cubn* loci (Fig. 5b, l). The formation of a direct chromatin–chromatin interacting loop has been treated as a common mechanism employed by genomic regulatory elements, such as enhancers, promoters. Genomic elements with enhancer activities are usually involved in the maintenance or upregulation of target gene expression. However, in our study, although direct chromatin interaction can be identified between *Gm26793* and *Cubn*, *Gm26793* seems to be a repressive molecular lock, which can silence *Cubn* expression in normal mESCs (Fig. 5c). By cross-referencing the published dataset, we found that the *Gm26793* locus also harbors H3K27me3 and the PRC2 complex distribution (Supplementary Fig. S10f). This suggests that *Gm26793* may operate through a mechanism similar to recently identified regulatory elements known as silencers^103^. A more comprehensive characterization of epigenetic features, sequence composition as well as the nuclear localization of *Gm26793* and its transcripts will facilitate the understanding of how this molecular lock works to silence *Cubn* expression.

We identify CTCF as the crucial architectural mediator that establishes the inter-chromosomal lock between *Gm26793* and *Cubn*. As shown in Fig. 7c, we find that CTCF specifically binds to the genomic locus of *Gm26793* and *Cubn*. Genetic removal of individual CTCF coupling anchor site (CBS-KO or CKO) significantly disrupts the inter-chromosomal interaction, further resulting in the boosted responsiveness to primitive endoderm differentiation signals (Fig. 7d–k). As is known, CTCF has been recognized as one of the fundamental players in the “loop extrusion” model within the same chromosome, especially for the establishment of TADs^104^. Since the specific interaction occurs between two distinct chromosomes in this study, it remains to determine whether CTCF behaves similarly as in intra-chromosomal interaction, in composing the “inter-chromosomal genomic love story of kissing”^105^. As revealed by the epigenomic profiling of H3K27ac, H3K4me3, and chromatin accessibility, a single knock-out of *Gm26793* or CKO locus leads to global pervasive remodeling of the epigenetic landscape (Fig. 6b, g; Supplementary Fig. S10d, e). Accompanying complex molecular cascades possibly participate in the response to the genetic alteration. Thus, we hypothesize that additional factors, like epigenetic factors, may exist in concert with CTCF in manipulating this inter-chromosomal interaction. Further investigation of the epigenetic features and motif enrichment will provide valuable insights into the precise mechanisms of this molecular lock.

In conclusion, our observation extends the classical paradigm of how transcriptional regulation occurs through lncRNA, and reveals the existence and biological significance of inter-chromosomal interaction during stem cell differentiation and embryo development. Future studies related to molecular dynamics reflecting the formation and maintenance of inter-chromosomal interactions and subsequent molecular cascades will broaden the horizon of stem cell fate determination and mammalian embryogenesis.

## Materials and Methods

### Mouse strains

All mice were housed in individually ventilated cages under specific pathogen-free conditions and handled according to the guidelines of the Animal Ethical Committee of the Institute of Biochemistry and Cell Biology, Shanghai Institutes for Biological Sciences, Chinese Academy of Sciences. To generate GKO mice, we firstly derived GKO DKO-AG-haESCs by CRISPR-Cas9. Then GKO female mice were constructed via intracytoplasmic AG-haESCs injection (ICAHCI)^106^ and bred with WT males (C57BL/6 J) to produce GKO heterozygous offspring. The heterozygous mice were mated internally to generate GKO homozygous mice. WT embryos were collected from the C57BL/6 J background mice.

### Embryos

For preimplantation embryos, all zygotes were obtained from superovulated and fertilized female mice, and then cultured in EmbryoMax Advanced potassium-supplemented simplex optimized medium (KSOM) with amino acids under mineral oil on polystyrene plates. Embryos were maintained in a humidified incubator at 37 °C with 5% CO_2_ until the blastocyst stage. For gastrula, embryos were removed from the implantation site as described previously^107^. Briefly, plugged female mice were picked after mating and counted as E0.5. Mice were euthanized when embryos developed into nominal day 7.5. The embryos were acquired through the removal of the surrounding decidua and Reichert’s membrane by using sharpened surgeon tweezers.

### mESCs culture and differentiation

mESCs (E14) were cultured under feeder-free conditions on gelatinized dishes in DMEM medium supplemented with 15% FBS, 1% GlutaMAX, 1% NEAA, 1 mM sodium pyruvate, 0.1 mM β-mercaptoethanol, 1% penicillin/streptomycin, 1000 U/mL mouse LIF, 3 μM CHIR99021, 1 μM PD0325901, and passaged by single-cell trypsinization every 2–3 days. For EB differentiation, mESCs were dissociated with 0.05% trypsin and suspended in differentiation medium consisting of DMEM, 10% FBS, 1% GlutaMAX, 1% NEAA, 1 mM sodium pyruvate, 0.1 mM β-mercaptoethanol and 1% penicillin/streptomycin. 1 × 10^5^ cells/mL were plated in Petri-dishes and cultured for 8 days. Every 2 days, we changed the medium and divided the differentiated EBs into fresh Petri-dishes. For XEN differentiation, 1 × 10^4^ cells/cm^2^ were seeded on gelatin-coated dishes and cultured in standard XEN medium consisting of RPMI-1640, 15% FBS, 1% GlutaMAX, 1% penicillin/streptomycin, and 0.1 mM β-mercaptoethanol for 1 day, then the medium was changed to derivation XEN medium (standard XEN medium supplemented with 0.01 μM RA and 10 ng/mL Activin A). After two days of culture, differentiated cells were dissociated into single cells and plated at a 1:1 ratio on MEF-coated dishes, and thereafter maintained in standard XEN medium.

### CRISPR-Cas9-mediated knockout and knockin

The deletion of the *Gm26793* locus and CTCF-binding sites in mESCs was performed by CRISPR-Cas9 gene editing system. Briefly, a pair of sgRNAs (upstream and downstream) was designed and inserted into the pX330-mCherry vector. mESCs were transfected with 5 μg sg-up&down plasmids by Lipofectamine^TM^ 2000 and cultured for 24 h. 1 × 10^4^ mCherry-positive single cells were sorted to seed on a 10-cm gelatinized dish. After 4–6 days of culture, individual colonies were picked up and expanded in 48-well plates. Finally, genomic deletion mESCs were validated by PCR and Sanger sequencing. sgRNA oligos and genotyping primers were presented in Supplementary Tables S13 and S14. Regarding gene insertion, the genomic region flanking the knockin site and introduced sequence for *Gm26793* were PCR amplified and mixed with linearized pGEM-T plasmids, and annealed to generate the donor vector. Then, the donor and sgRNA plasmids were transfected into the mESCs according to the concentration ratio of the knockout strategy. Subsequent cell sorting and genotyping were performed as described above.

### RNAi and overexpression assays

For knockdown experiments, shRNAs were constructed into lentiviral vector pLKO.1 for lentiviral packaging. After 48 h transfection into mESCs, puromycin-resistant cells were selected for testing the efficiency of knockdown. The sequence of shRNAs targeting specific genes was designed by using the online tool GPP Web Portal (https://portals.broadinstitute.org/gpp/public/) and is listed in Supplementary Table S14. For overexpression experiments, ORFs were similarly cloned into lentiviral vector Fugw-IRES-dsRed or Fuw-TRE-P2A-mCherry (inducible). RFP-positive mESCs were eventually sorted for functional analysis. The inducible overexpression system will proceed under the treatment of Dox.

### Whole-mount in situ hybridization

Digoxigenin (DIG)-labeled riboprobes were synthesized as previously reported^108^. Primers used for amplifying probe templates are listed in Supplementary Table S13. In brief, E7.5 embryos were fixed with 4% PFA, dehydrated and rehydrated through 100%, 75%, 50%, and 25% methanol. Samples were then treated with 10 μg/mL proteinase K for 10 min and post-fixed with 0.1% glutaraldehyde for 30 min. Approximately 1 μg/mL DIG-labeled RNA-probe was incubated with the embryos at 70 °C overnight. After washing, the embryos were incubated in anti-DIG-AP at 4 °C overnight, then washed and stained with NBT and BCIP for imaging.

### qPCR

Total RNA was extracted from cultured cells by using TRIZOL reagent and 500 ng to 2 μg RNA was reverse transcribed into first-strand cDNA by FastKing RT Kit (Tiangen, KR116). qPCR analysis was performed with Mastercycler Realplex2 (Eppendorf) using Stormstar SYBR green qPCR master mix (DBI-2144). The relative expression of target genes was normalized to the internal control *Gapdh* and quantified by 2^–ΔΔCt^ methods. qPCR primers for specific genes are presented in Supplementary Table S13.

### Immunostaining

After fixation with 4% PFA for 30 min, the cultured cells were permeabilized and blocked with 0.3% Triton X-100/5% BSA in PBS for 1 h at room temperature. Then, the samples were incubated with primary antibodies (1:200) at 4 °C overnight. The next day, samples were washed 3 times and incubated with fluorescence-conjugated secondary antibodies (1:500–1:1000) for 1 h at room temperature. The nuclei were stained with DAPI (1:1000). For staining of embryoid bodies, samples were dehydrated in 20% sucrose at 4 °C overnight after fixation, and then embedded in OCT for cryosection. Slides immunostaining was conducted as described above. The antibody information is listed in Supplementary Table S16 and images were taken using Leica TCS SP8 confocal laser-scanning microscope.

### Western blotting

The harvested cells were lysed in RIPA buffer with protease and phosphatase inhibitors for 30 min on ice. After centrifugation, proteins in the supernatant were quantified and added to the loading buffer for heating 10 min at 100 °C. 20 ug total protein was separated by SDS-PAGE and transferred to PVDF membranes. The membranes were blocked with 5% BSA and incubated with primary antibodies (1:1000) at 4 °C overnight. After three times of washing with TBST, the membranes were incubated with HRP-conjugated secondary antibodies (1:2000) for 1 h at room temperature, and the target proteins were detected by SuperSignal™ West Pico PLUS Substrate (Thermo Fisher Scientific, 34580) subsequently.

### Geo-seq of EB samples

Geo-seq was performed as previously described^69^. Briefly, whole EBs were embedded in OCT and cryosectioned at a thickness of 20 μm. Sections were mounted on polyethylene-terephthalate-coated slides, fixed with ethanol, and stained with 1% DAPI in 75% ethanol solution. Then, ~20 cells in the designated region were captured by laser microdissection (MMI Cellcut Plus system) and lysed in 50 μL 4 M guanidine isothiocyanate for 15 min at 42 °C. After isolation through ethanol precipitation, dissolved RNA was immediately reversely transcribed into cDNA and amplified by Smart-seq2.

### CRISPR-dCas9-based imaging

To visualize *Gm26793*–*Cubn* loci interaction, we used the CRISPR-mediated DNA labeling system to achieve non-repetitive DNA imaging, according to the previous description^93^. Briefly, 30 bp crRNAs targeting *Gm26793* and *Cubn* loci were synthesized with fluorescent labeling (Cy5-Gm26793; TAMRA-Cubn) at the 5′-end (Sango). The sequences of crRNAs used in this study are presented in Supplementary Table S14. The crRNAs and tracrRNA were annealed and incubated with dCas9 protein (IDT) to form fluorescent RNA protein complexes (fRNPs). Then, mESCs were transfected with the pre-assembled fRNP pool by electroporation (program: OP315 or CD112) using an SE Cell line 4D-Nucleofector™ X kit (Lonza, V4XC-1024). The electroporated cells were plated in Nunc Glass Bottom Dishes (Thermo Fisher Scientific, 150680) and cultured for 12–24 h before imaging. Microscopic imaging was performed on a Leica DMi8 Inverted Microscope using sCMOS camera and APO 63×/1.4 oil objective or a Leica TCS SP8 STED equipped with the spectral flexibility of WLL for excitation and an HC PL APO 100×/1.4 oil objective with *Z*-stacks from 0.27 to 5 μm. Nuclei were visualized using DAPI for fixed cells or NucBlue™ Live ReadyProbes™ (Thermo Fisher Scientific, R37605) for living cells.

### Image analysis

To quantify the 3D distance between *Gm26793* and *Cubn* loci, the imaging data were processed using deconvolution wizard and chromatic Aberration Corrector to generate the ICS2 files after adjusting imaging parameters of channel (excitation and emission wavelengths), type of microscope, material of vehicle and imaging optical path media, automatically generated theoretical PSF and correct *Z*-drift in Huygens software. The ICS2 files were loaded by Imaris to measure the distance between *Gm26793*–*Cubn* loci after performing spot simulation based on each fluorescence channel maximum value corresponding to the *Gm26793*–*Cubn* loci.

### Tn5-FISH

Tn5-FISH was performed according to a previously reported (Tn5-FISH)^109^. Briefly, the probe library was generated by PCR amplification and recovered using a DNA Cleanup kit (TIANGEN, DP203-02). After recovering using the DNA Cleanup kit, salmon sperm DNA (Invitrogen, 18440016) was added into the Tn5-FISH probes (50 mg of salmon sperm DNA per 1 mg of Tn5-FISH probes), ethanol precipitated, and dissolved in DNA FISH buffer, 50% deionized formamide (Ambion, AM9342), 10% dextran sulfate (VWR, 9011-18-1), 2× SSC (Invitrogen, 15557044), at a concentration of 20 ng/uL of Tn5-FISH probes. The Tn5-FISH probes were amplified by a second PCR with fluorescence-tagged primers. The in situ hybridization procedure of Tn5-FISH was similar to that of traditional FISH, as previously described^110^. Microscopic imaging was performed on Dragonfly200 or a Leica TCS SP8 STED equipped with the spectral flexibility of white-light laser for excitation and an HC PL APO 100×/1.4 oil objective. The sequences of the utilized primers are presented in Supplementary Table S15.

### Bulk RNA-seq data processing and analysis

Data quality control was performed to ensure the reliability of the results. Quality control metrics such as sequence quality scores, GC content, and adapter content were assessed using FastQC (v0.11.9). Low-quality reads and adapters were trimmed or removed. Then, the high-quality reads were aligned to a reference genome Hisat2 (v2.2.1), and the reference genome used was mm10. Gene-level expression quantification was performed using featureCounts (v1.5.3). This step assigns reads to genes and generates a count matrix representing the number of reads mapped to each gene in each sample. The count matrix was normalized to account for differences in sequencing depth and gene length as fragments per kilobase of transcript per million mapped reads (FPKM). Differential expression analysis was performed to identify genes that were differentially expressed between conditions or groups of interest using the DESeq2 package with a count matrix as input.

### Annotation and identification of DELs during mouse gastrulation

Raw lncRNA-seq data were obtained from GEO-seq datasets^63^. The high-quality reads were aligned to a reference genome using the read aligner Tophat2. The reference genome used was mm10. An annotation file containing known lncRNA transcripts was obtained from databases such as GENCODE. The alignment results were filtered to retain only reads that mapped to the annotated lncRNA regions. Quantification of lncRNA expression levels was performed with Cufflinks. This step assigns reads to lncRNA transcripts and generates an FPKM matrix. DELs were identified as follows: (1) calculation of the variance of each expressed lncRNA across all samples and selection of top ~1000 genes as highly variable genes; (2) hierarchical clustering with correlation distance metric based on *z*-score normalized expression of highly variable genes to identify preliminary domains according to distinctly separated dendrogram; (3) identification of the inter-domain DELs, based on expression of highly variable genes by pairwise comparisons of preliminary domains using *t*-test (*P* < 0.05) and fold change (FC >= 1.5); (4) combination of top highest and lowest principal component (PC)-loading genes (by using FactoMineR (2.8) in R) from several selected significant PCs by jackstraw to identify the DELs (top 300 genes for each of PC1–4). Finally, K-means clustering was applied to determine the final spatial domains of the embryo based on the expression profile of DELs, and the BIC-SKmeans algorithm was applied to determine the optimal number of gene groups and perform gene clustering analysis based on the *z*-score normalized expression profile of DELs. The clustering heatmap was visualized through ComplexHeatmap (2.15.1).

### WGCNA

Co-expression networks were constructed using WGCNA (v 1.72.1) package in R^68^. Firstly, we created a matrix of pairwise correlations between all pairs of genes across the measured samples. Next, we identified the soft thresholding power (β) value based on the scale-free topology network criterion and converted the expression matrix into an adjacency matrix. The adjacency matrix was then transformed into a topological overlap matrix (TOM) to capture the interconnectedness of genes within the network. Next, the topological overlap dissimilarity was calculated using TOM, followed by hierarchical clustering to identify separated gene modules. A dynamic tree-cutting algorithm was employed for gene module determination, with a minimum size of 30, and highly similar modules would be merged automatically. Then, the module eigengene, which represents the first PC of each module, was estimated and summarizes the overall expression pattern of genes within one module. Finally, we can perform module-trait relationship analysis to assess the correlation coefficient between module eigengenes and sample traits or phenotypes of interest. This analysis helps to identify certain unique gene modules associated with specific biological conditions.

### 4C-seq analysis

4 C was carried out by a modified published protocol^111^. In brief, 5 ×10^6^ mESCs were fixed in 1% formaldehyde solution and quenched with 0.125 M glycine, then rinsed by DPBS twice before being frozen in liquid nitrogen. After thawing on ice, fixed cells were resuspended in lysis buffer (50 mM Tris-HCl pH 7.5, 0.5% NP-40, 1% Triton X-100, 150 mM NaCl, 5 mM EDTA) and 0.5% SDS buffer for 30 min, respectively. Each sample was then permeabilized in 1% Triton X-100 and digested with *Dpn*II for 3 h at 37 °C. After the first ligation, fragmented DNA was purified by using phenol-chloroform and ethanol precipitation upon treatment with RNase A and Proteinase K, then digested again with *Csp*6I for 3 h at 37 °C. After the second ligation, circularized DNA was purified with AMPure XP beads, and 4C-libraries were finally generated by two rounds of PCR and purified by QIAGEN column. The first PCR step was conducted to reversely amplify the DNA fragments ligated to the *Gm26793* viewpoint. The second PCR step was to add an Illumina index for high-throughput sequencing. Primers used for 4 C library construction are presented in Supplementary Table S14. Proteinase inhibitor cocktail and PMSF are required to be added before the second digestion to prevent protein degradation. Three biological replicates were performed for 4C-seq analysis of the *Gm26793* locus. Sequencing reads with 5’-end matching the inverse PCR primer sequence were selected and trimmed, remaining sequences containing *Dpn*II and *Csp*6I sites were mapped to mm10 assembly using Bowtie2 (v2.4.5), and the interaction regions were identified based on the pipeline proposed by Krijger et al^111^.

### ChIP-seq analysis

ChIP was performed as previously described^112^. Briefly, cross-linked mESCs were lysed in lysis buffer and fragmented to a size range of 200–500 bp by using Bioruptor Pico. Then, solubilized fragmented chromatin was immunoprecipitated with primary antibodies (CTCF, RAD21, H3K27ac, and H3K4m3) and pulled down by protein G beads. Reverse crosslink was performed at 65 °C for at least 4 h. Subsequently, ChIP-DNA was treated with RNase A and Proteinase K, precipitated with ethanol, and dissolved in nuclease-free water. Proteinase inhibitor cocktail and PMSF were added in all immunoprecipitation assays to inhibit protein degradation. Additionally, 20 mM sodium butyrate was added in H3K27ac group to inhibit histone deacetylase. Finally, ChIP libraries were prepared by using NEBNext® Ultra™ DNA Library Prep Kit (NEB, E7770L) for Illumina. ChIP-seq reads were mapped to the mm10 genome with Bowtie2 (v2.4.5) using default parameters, then the peaks were called using MACS2 (v2.2.7) to identify regions of the genome that exhibit significant enrichment for CTCF, RAD21, and histone modifications compared to background. Differential peaks of ChIP-seq experiments were called with the R package, DiffBind (v3.6.5), under default settings. Heatmaps of ChIP-seq signal enrichment were generated by the Python package, deepTools (v3.5.1). Annotation of ChIP-seq peaks was done by ChIPseeker (v1.32.1).

### ATAC-seq analysis

For ATAC-seq, 5 × 10^4^ cells were harvested and resuspended in lysis buffer (10 mM Tris-HCl pH 7.4, 0.15% NP-40, 10 mM NaCl, 3 mM MgCl_2_). After vortexing 3 times every 3 min, the tube was centrifuged at 1000× *g* for 10 min at 4 °C, and the supernatant was discarded. Then, the cell pellet was resuspended in fragmentation buffer (5× TTBL, TTE V50 Mix) (Vazyme, TD501) and incubated at 37 °C for 30 min. Immediately, fragmented DNA was purified by QIAGEN MinElute Kit, and sequencing libraries were generated by PCR using NEB Q5 Master mix (E7649A). The size of the library was selected and purified with AMPure XP beads. The package versions involved in ATAC-seq data processing are the same as those in ChIP-seq analysis.

### Blastocyst collection and single-cell isolation

To obtain adequate blastocysts, WT and GKO female mice (8–10 weeks) were induced to superovulate by injection of 7.5 IU pregnant mare’s serum gonadotropin (PMSG) followed by 7.5 IU human chorionic gonadotropin (hCG), and then were mated with male mice. Vaginal plugs were checked the next morning. Zygotes were collected from the oviduct 13–15 h after hCG injection. Embryos were cultured with KSOM medium containing amino acids in an incubator with 5% CO_2_ at 37 °C. Early and late blastocysts were collected at E3.5 and E4.5, respectively. Single-cell isolation of blastocysts was performed as follows. After removal of zona pellucida using acid Tyrode solution, the embryos were dissociated with 1% trypsin/EDTA for 30–40 min at 37 °C, then washed twice and resuspended in 0.1% BSA/PBS. Single cells were manually picked into PCR tubes by mouth pipette under a microscope. For the collection of scRNA-seq samples at E3.5, we ruled out the arrested embryos with aberrant cleavage before E3.5. As to E4.5, we mixed normal and abnormal GKO blastocysts for scRNA-seq.

### scRNA-seq analysis

Single cells were lysed in lysis buffer containing 0.45% NP-40, followed by reverse transcription using SuperScript II reverse transcriptase. Subsequently, the entire cDNA was amplified with 2× KAPA Mix and PCR products were purified with AMPure XP beads and quantified with Qubit. cDNA libraries were then constructed with TruePrep DNA Library Prep Kit V2 (Vazyme, TD503) for Illumina. In total, 1007 single cells were sequenced for further analysis. Library construction and purification were completed on the Agilent Bravo automatic liquid-handling platform. Raw counts of genes were calculated for each cell using the same workflow as for bulk RNA-seq. Seurat (v4.3.0) was utilized for the analysis of single-cell data. Mapping and annotation of query datasets were performed referring to this workflow (https://satijalab.org/seurat/articles/integration_mapping.html). Briefly, UMAP was applied to visualize the cell clusters in two dimensions. Clustering was performed based on the shared nearest neighbor graph using algorithms such as Louvain clustering or density-based clustering. Cell type annotation was performed by comparing the cluster-specific marker genes with known cell type marker genes from databases or literature. Functional enrichment analysis, such as GO or pathway enrichment, was performed with Metascape to identify the biological processes, molecular functions, and pathways enriched in specific cell clusters. Monocle 3^81^ (1.2.9) was applied to reconstruct the pseudotime trajectories. We manually selected the inner cell mass as the root node and used variable genes identified in Seurat as ordering genes in the Monocle 3 pipeline. Monocle 3 used a graph-embedding algorithm to learn a trajectory that fits the UMAP-coordinated cell clusters.

### Quantification and statistical analysis

For quantification of the immunostaining, we counted the distribution of corresponding antibody-positive cells in five randomly selected visual fields. All experiments were performed with at least three biological replicates. Student’s *t*-tests were used to compare the effects of the two different groups. One way- and Two way-ANOVA tests were used to compare the effects of different three or more groups. Differences were considered statistically significant at *P < 0.05, **P < 0.01, ***P < 0.001. GraphPad Prism 8 and Excel were used for statistical calculations and generation of plots.

## Supplementary information

Supplementary Figures 1-10 and Legends

Supplementary Table 1

Supplementary Table 2

Supplementary Table 3

Supplementary Table 4

Supplementary Table 5

Supplementary Table 6

Supplementary Table 7

Supplementary Table 8

Supplementary Table 9

Supplementary Table 10

Supplementary Table 11

Supplementary Table 12

Supplementary Table 13

Supplementary Table 14

Supplementary Table 15

Supplementary Table 16

## Data Availability

The raw sequence data reported in this manuscript have been deposited in the Genome Sequence Archive in the National Genomics Data Center, China National Center for Bioinformation/Beijing Institute of Genomics, Chinese Academy of Sciences with the accession numbers of GSA: CRA011812, CRA011818, CRA011832, CRA011839, CRA011986, CRA018127 and CRA018128 that are publicly accessible at https://ngdc.cncb.ac.cn/gsa.

## Code availability

The raw sequence data reported in this manuscript have been deposited in the Genome Sequence Archive in the National Genomics Data Center, China National Center for Bioinformation/Beijing Institute of Genomics, Chinese Academy of Sciences with the accession numbers of GSA: CRA011812, CRA011818, CRA011832, CRA011839, CRA011986, CRA018127 and CRA018128 that are publicly accessible at https://ngdc.cncb.ac.cn/gsa.
