# Supplementary material for: Genomic locus of lncRNA-Gm26793 forms an inter-chromosomal interaction with Cubn to ensure proper stem cell differentiation in vitro and in vivo
Source: Cell Discov. 2025 Jun 3;11:53. doi: 10.1038/s41421-025-00805-0 (PMC12134126; doi:10.1038/s41421-025-00805-0)
Supplement: Supplementary file 1 — Supplementary Figures 1-10 and Legends [file 41421_2025_805_MOESM1_ESM.pdf]

Fig. S1

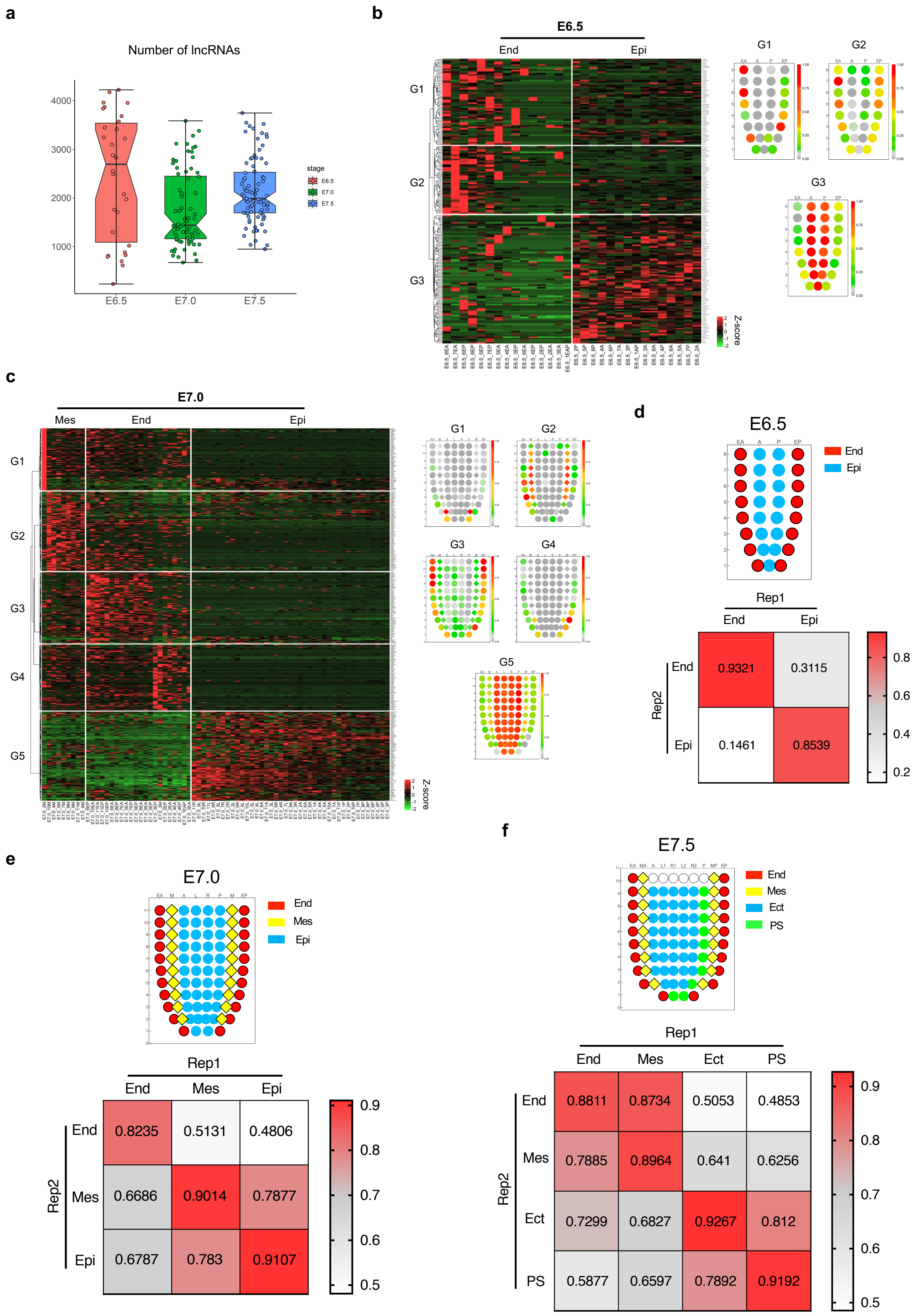
a
Number of lncRNAs
4000
3000
2000
1000
E6.5
E7.0
E7.5
stage
E6.5
E7.0
E7.5
b
E6.5
End
Epi
G1
G2
G3
Z-score
c
E7.0
Mes
End
Epi
G1
G2
G3
G4
G5
Z-score
d
E6.5
End
Epi
Rep1
End
Epi
Rep2
End
0.9321
0.3115
Epi
0.1461
0.8539
0.8
0.6
0.4
0.2
e
E7.0
End
Mes
Epi
Rep1
End
Mes
Epi
Rep2
End
0.8235
0.5131
0.4806
Mes
0.6686
0.9014
0.7877
Epi
0.6787
0.783
0.9107
0.9
0.8
0.7
0.6
0.5
f
E7.5
End
Mes
Ect
PS
Rep1
End
Mes
Ect
PS
Rep2
End
0.8811
0.8734
0.5053
0.4853
Mes
0.7885
0.8964
0.641
0.6256
Ect
0.7299
0.6827
0.9267
0.812
PS
0.5877
0.6597
0.7892
0.9192
0.9
0.8
0.7
0.6
0.5

Fig. S1 Differentially expressed lncRNAs during mouse gastrulation.

(a) Box plot showing the number of detected lncRNAs for each GEO-seq samples from E6.5 to E7.5 mouse embryos.

(b-c) Heatmaps and corn plots of lncRNAs with germ layer specific expression in E6.5 (b) and E7.0 (c) gastrula. End: endoderm; Mes: mesoderm; Epi: epiblast; A: anterior epiblast; P: posterior epiblast; L: left lateral epiblast; R: right lateral epiblast; EA: anterior endoderm; EP: posterior endoderm; M: whole mesoderm.

(d-f) The spearman correlation coefficient of distinct germ layers between embryonic replicates at E6.5 (d), E7.0 (e) and E7.5 (f) stage based on the identified differentially expressed lncRNAs.

Fig. S2

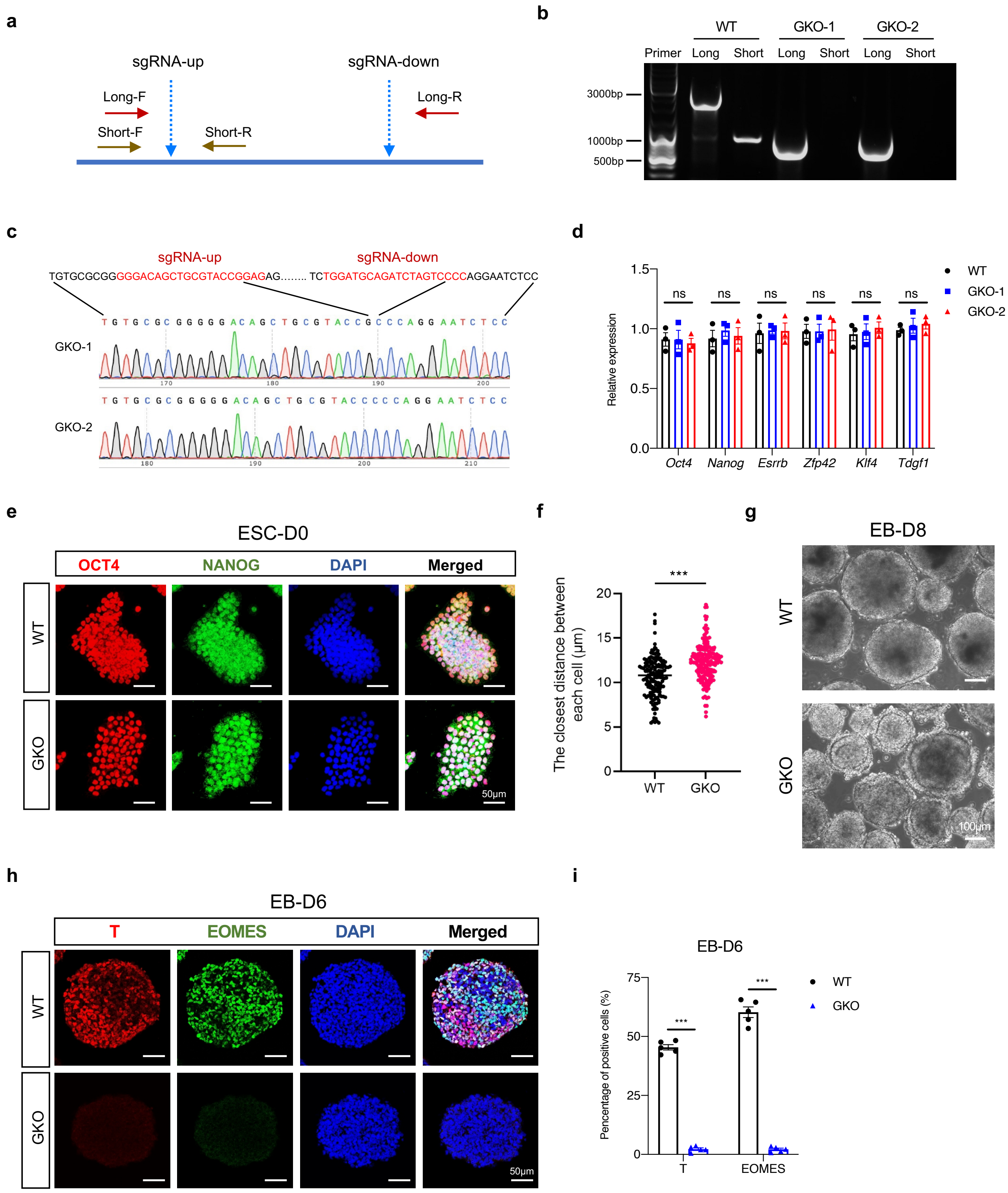

a
sgRNA-up
sgRNA-down
Long-F
Long-R
Short-F
Short-R
b
WT
GKO-1
GKO-2
Primer
Long
Short
Long
Short
Long
Short
3000bp
1000bp
500bp
c
sgRNA-up
sgRNA-down
TGTGCGCGGGGGACAGCTGCGTACCGGAGAG........ TCTGGATGCAGATCTAGTCCCCAGGAATCTCC
GKO-1
GKO-2
d
Relative expression
ns
WT
GKO-1
GKO-2
Oct4
Nanog
Esrrb
Zfp42
Klf4
Tdgf1
e
ESC-D0
OCT4
NANOG
DAPI
Merged
WT
GKO
50μm
f
The closest distance between each cell (μm)
***
WT
GKO
g
EB-D8
WT
GKO
100μm
h
EB-D6
T
EOMES
DAPI
Merged
WT
GKO
50μm
i
EB-D6
Percentage of positive cells (%)
***
WT
GKO
T
EOMES

Fig. S2 The establishment of GKO mESCs and expression identification of mesoderm-related genes during EB differentiation.

(a) Schematic description of genotyping strategy and related primer designed in determining knockout clones.

(b) Representative image of gel electrophoresis showing the genotyping PCR results of GKO mESCs.

(c) Sanger sequencing results of the acquired GKO cells demonstrating successful removal of targeted allele in *Gm26793* locus.

(d) Bar plot showing the comparable expression level of core pluripotency marker genes in WT and GKO mESCs.

(e) Co-immunostaining analyses of OCT4 and NANOG in WT and GKO mESCs. Scale bar, 50 μm.

(f) Statistic calculation of the spatial distance between each nucleus in WT and GKO mESCs.

(g) The morphologies of differentiated EBs at day 8 in both WT and GKO group. Scale bar, 100 μm.

(h-i) Immunoflorescence analyses showing the distribution of T and EOMES protein in WT and GKO EBs collected at day 6. Scale bar, 50 μm. Quantification of the data is shown in (i).

All data are shown as means ± SEM. Student's t-test analysis is used in f, i; *** p<0.001.

Fig. S3

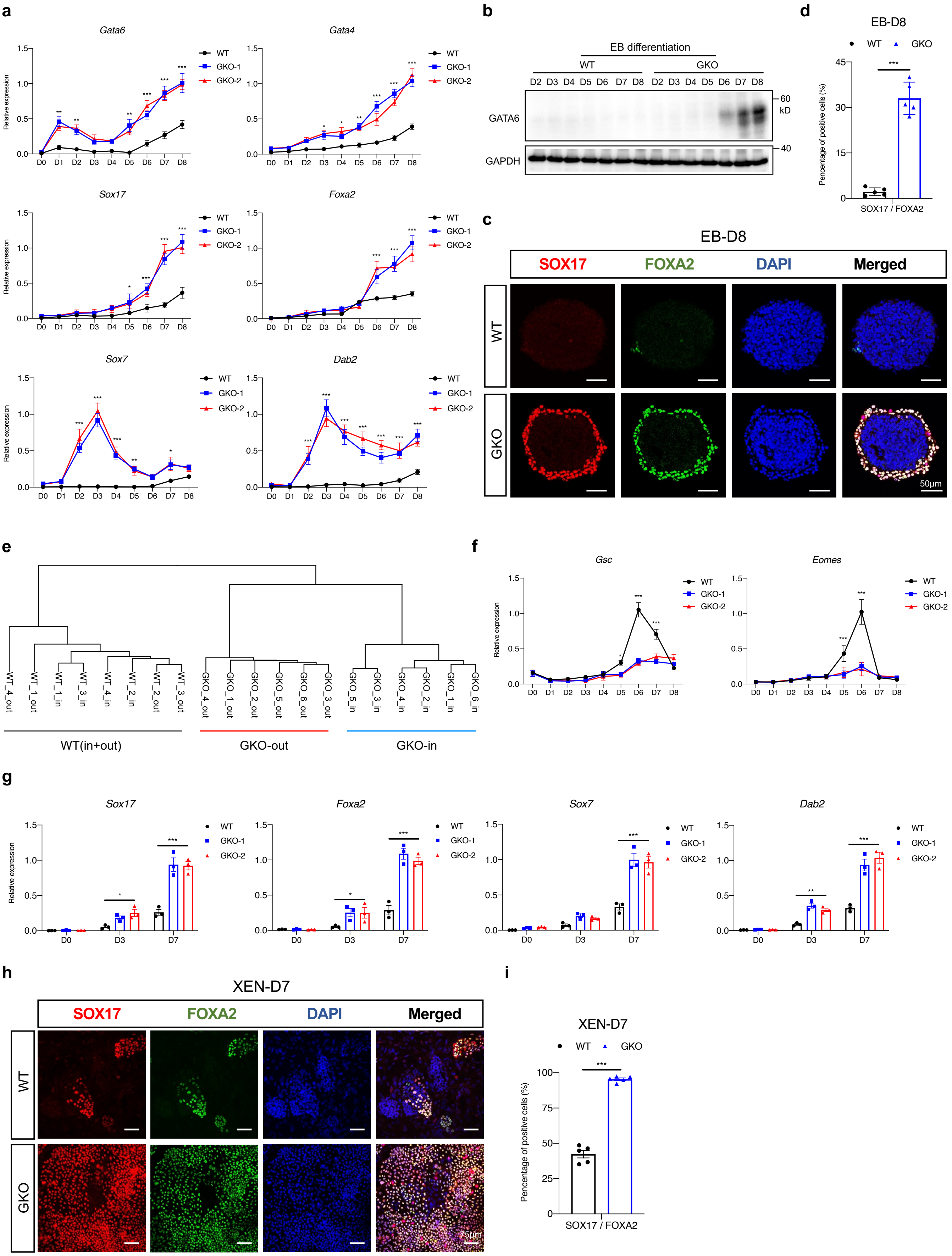

a
Gata6
Gata4
Sox17
Foxa2
Sox7
Dab2
WT
GKO-1
GKO-2
Relative expression
D0 D1 D2 D3 D4 D5 D6 D7 D8
b
EB differentiation
WT
GKO
D2 D3 D4 D5 D6 D7 D8 D2 D3 D4 D5 D6 D7 D8
GATA6
GAPDH
60
kD
40
d
EB-D8
WT
GKO
Percentage of positive cells (%)
SOX17 / FOXA2
c
EB-D8
SOX17
FOXA2
DAPI
Merged
WT
GKO
50μm
e
WT_4_out
WT_1_out
WT_1_in
WT_3_in
WT_4_in
WT_2_in
WT_2_out
WT_3_out
GKO_4_out
GKO_1_out
GKO_2_out
GKO_5_out
GKO_6_out
GKO_3_out
GKO_5_in
GKO_3_in
GKO_4_in
GKO_2_in
GKO_1_in
GKO_6_in
WT(in+out)
GKO-out
GKO-in
f
Gsc
Eomes
g
Sox17
Foxa2
Sox7
Dab2
D0
D3
D7
h
XEN-D7
SOX17
FOXA2
DAPI
Merged
WT
GKO
75μm
i
XEN-D7
WT
GKO
Percentage of positive cells (%)
SOX17 / FOXA2

Fig. S3 The expression of PrE marker genes were largely enhanced in GKO cells during EB and XEN differentiation.

(a) qPCR analyses identify the immediate activation of PrE-related genes during EB differentiation upon *Gm26793* knockout.

(b) Western blotting results reporting the aberrant activation of GATA6 in GKO cells during EB differentiation.

(c-d) Immunoflorescence analyses of SOX17/FOXA2 in WT and GKO EBs at day 8. Scale bar, 50 μm. Quantification of the data is shown in (d).

(e) Hierarchical clustering based on the transcriptomic data of outside and inside EB differentiation samples.

(f) The expression dynamics of mesendodermal markers-*Eomes* and *Gsc* during WT and GKO EB differentiation.

(g) Significant upregulation of PrE-related genes during XEN differentiation upon *Gm26793* knockout.

(h-i) Immunoflorescence analyses of SOX17/FOXA2 in WT and GKO XEN at day 7. Scale bar, 75 μm. Quantification of the data is shown in (i).

All data are shown as means ± SEM. Two-way ANOVA analysis with Tukey's test is used in a, f, g; Student's t-test analysis is used in d, i; *p<0.05, ** p<0.01, *** p<0.001.

Fig. S4

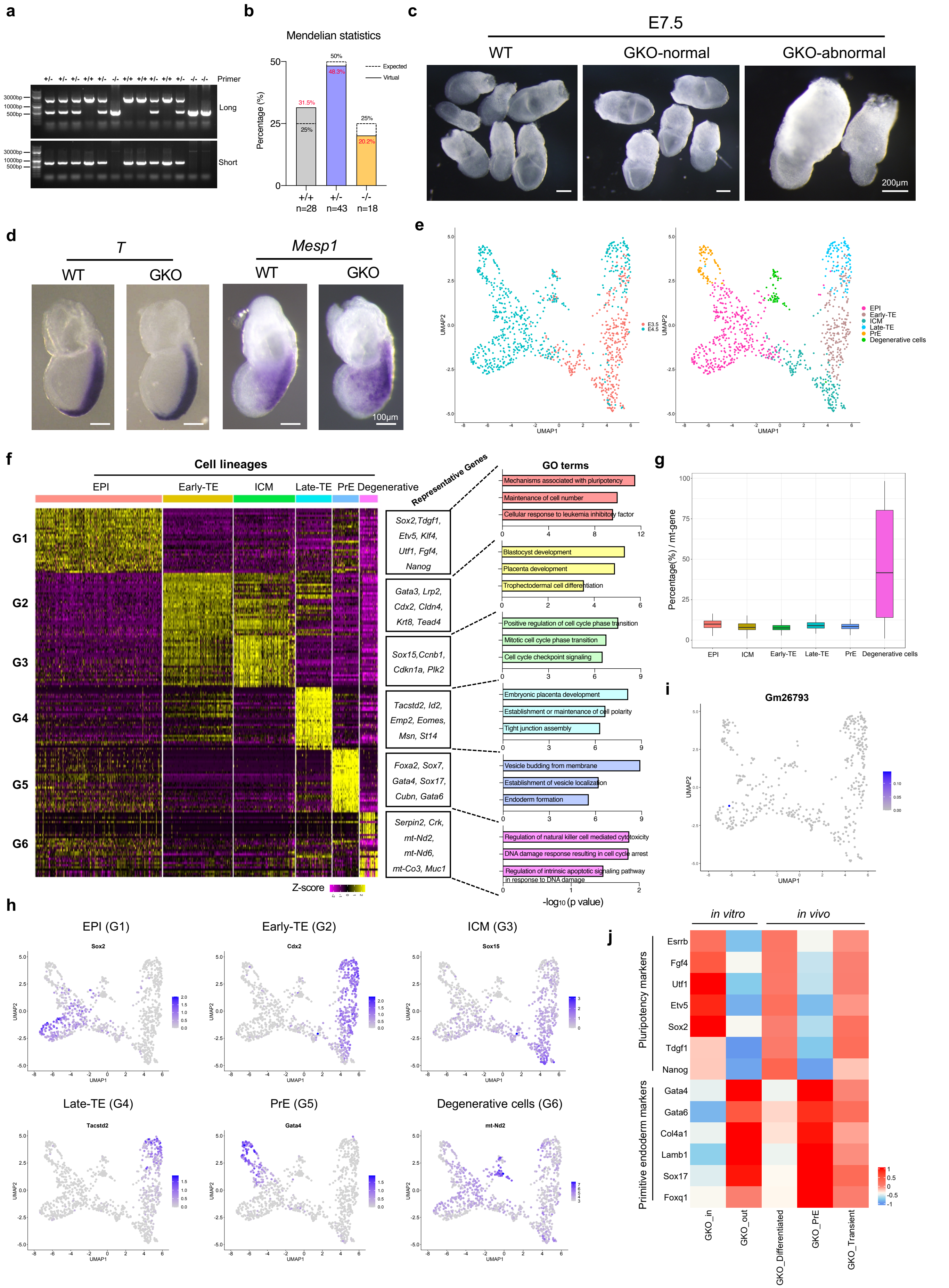

a
Primer
Long
Short
3000bp
1000bp
500bp
b
Mendelian statistics
Percentage (%)
Expected
Virtual
31.5%
25%
50%
48.3%
20.2%
+/+ n=28
+/- n=43
-/- n=18
c
E7.5
WT
GKO-normal
GKO-abnormal
200μm
d
T
Mesp1
WT
GKO
100μm
e
UMAP1
UMAP2
E3.5
E4.5
EPI
Early-TE
ICM
Late-TE
PrE
Degenerative cells
f
Cell lineages
EPI
Early-TE
ICM
Late-TE
PrE
Degenerative
Representative Genes
GO terms
G1
G2
G3
G4
G5
G6
Sox2,Tdgf1, Etv5, Klf4, Utf1, Fgf4, Nanog
Gata3, Lrp2, Cdx2, Cldn4, Krt8, Tead4
Sox15,Ccnb1, Cdkn1a, Plk2
Tacstd2, Id2, Emp2, Eomes, Msn, St14
Foxa2, Sox7, Gata4, Sox17, Cubn, Gata6
Serpin2, Crk, mt-Nd2, mt-Nd6, mt-Co3, Muc1
Mechanisms associated with pluripotency
Maintenance of cell number
Cellular response to leukemia inhibitory factor
Blastocyst development
Placenta development
Trophectodermal cell differentiation
Positive regulation of cell cycle phase transition
Mitotic cell cycle phase transition
Cell cycle checkpoint signaling
Embryonic placenta development
Establishment or maintenance of cell polarity
Tight junction assembly
Vesicle budding from membrane
Establishment of vesicle localization
Endoderm formation
Regulation of natural killer cell mediated cytotoxicity
DNA damage response resulting in cell cycle arrest
Regulation of intrinsic apoptotic signaling pathway in response to DNA damage
Z-score
-log10 (p value)
g
Percentage(%) / mt-gene
EPI
ICM
Early-TE
Late-TE
PrE
Degenerative cells
i
Gm26793
h
EPI (G1)
Sox2
Early-TE (G2)
Cdx2
ICM (G3)
Sox15
Late-TE (G4)
Tacstd2
PrE (G5)
Gata4
Degenerative cells (G6)
mt-Nd2
j
in vitro
in vivo
Pluripotency markers
Esrrb
Fgf4
Utf1
Etv5
Sox2
Tdgf1
Nanog
Primitive endoderm markers
Gata4
Gata6
Col4a1
Lamb1
Sox17
Foxq1
GKO_in
GKO_out
GKO_Differentiated
GKO_PrE
GKO_Transient

Fig. S4 The characterization of GKO embryos and cell type identification of scRNA-seq.

(a) DNA electrophoresis image showing the PCR genotyping of offspring born form GKO heterozygous mouse.

(b) Bar plot depicting the mendelian statistics of mouse progeny acquired through crossing GKO heterozygous mouse.

(c) Gross morphologies of WT embryos, GKO embryos with normal morphology, GKO embryos with abnormal morphology.

(d) Whole-mount in situ hybridization showing the indistinguishable distribution of *T* and *Mesp1* in WT and GKO embryos collected at E7.5 stage.

(e) UMAP plots of the gene expression profiles of individual cells regarding developmental stages (left) and cell types(right). The annotation for each cell cluster is included at the right side.

(f) Heatmap showing the expression of cell type-specific signature genes based on the single-cell transcriptome atlas. Representative genes and GO terms of each DEG group are listed in the right panel.

(g) The percentage of expressed mitochondrial genes in distinct cell populations.

(h) Expression profile of selected lineage-specific genes in UMAP plots.

(i) Expression profile of *Gm26793* in normal mouse blastocysts.

(j) Heatmap showing the expression levels of pluripotency and primitive endoderm marker genes across *in vitro* differentiation and *in vivo* lineage samples.

Fig. S5

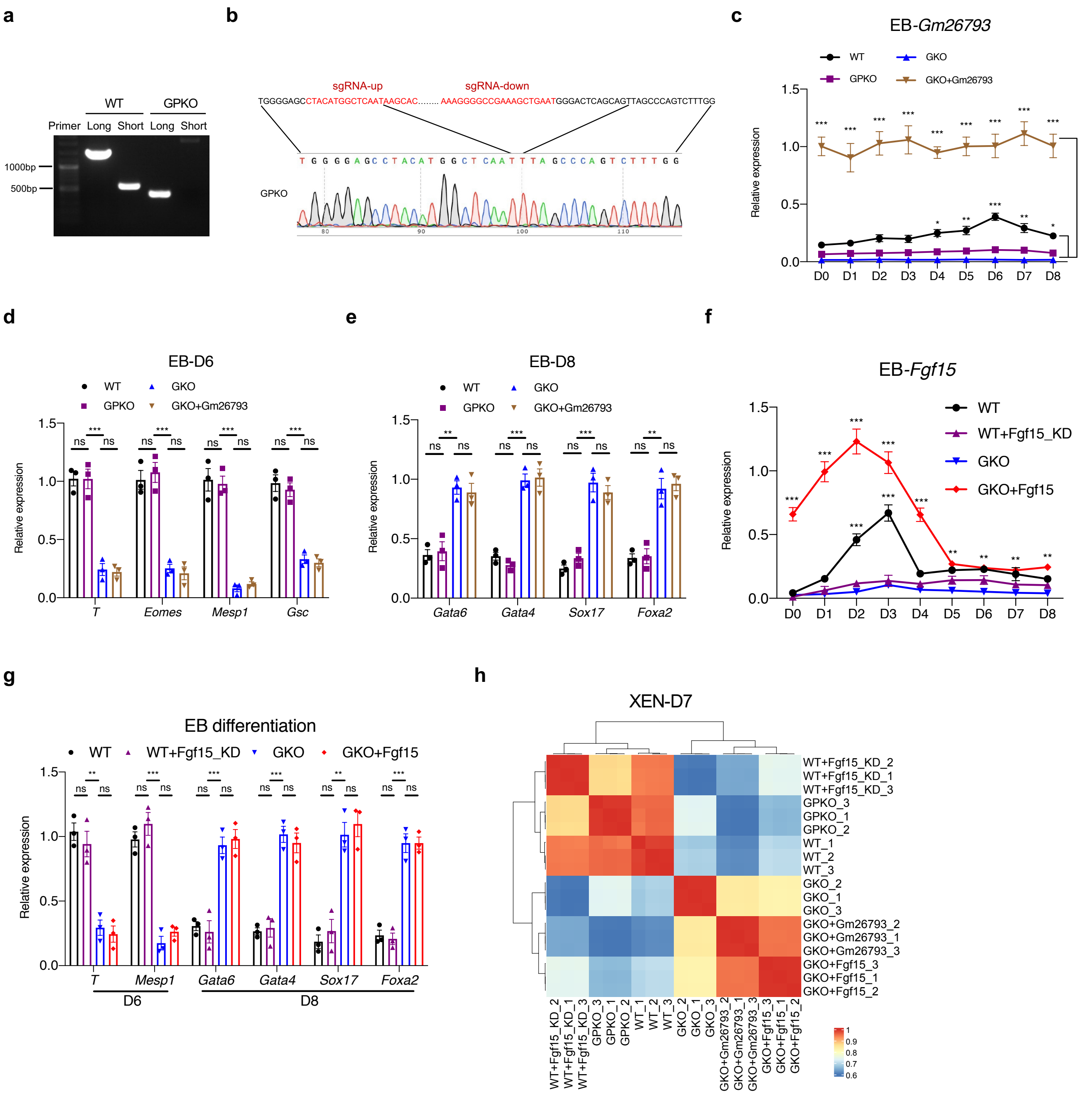
a
WT
GPKO
Primer
Long
Short
Long
Short
1000bp
500bp
b
sgRNA-up
sgRNA-down
GPKO
c
EB-Gm26793
WT
GKO
GPKO
GKO+Gm26793
Relative expression
D0
D1
D2
D3
D4
D5
D6
D7
D8
d
EB-D6
WT
GKO
GPKO
GKO+Gm26793
Relative expression
T
Eomes
Mesp1
Gsc
e
EB-D8
WT
GKO
GPKO
GKO+Gm26793
Relative expression
Gata6
Gata4
Sox17
Foxa2
f
EB-Fgf15
WT
WT+Fgf15_KD
GKO
GKO+Fgf15
Relative expression
D0
D1
D2
D3
D4
D5
D6
D7
D8
g
EB differentiation
WT
WT+Fgf15_KD
GKO
GKO+Fgf15
Relative expression
T
Mesp1
Gata6
Gata4
Sox17
Foxa2
D6
D8
h
XEN-D7
WT+Fgf15_KD_2
WT+Fgf15_KD_1
WT+Fgf15_KD_3
GPKO_3
GPKO_1
GPKO_2
WT_1
WT_2
WT_3
GKO_2
GKO_1
GKO_3
GKO+Gm26793_2
GKO+Gm26793_1
GKO+Gm26793_3
GKO+Fgf15_3
GKO+Fgf15_1
GKO+Fgf15_2

Fig. S5 Transcription of *Gm26793* and *Fgf15* is dispensable for primitive endoderm differentiation.

(a) DNA electrophoresis result demonstrating the removal of promoter region of *Gm26793* in mESCs.

(b) The genomic sequence of GPKO is confirmed by Sanger sequencing.

(c) The expression dynamics of *Gm26793* in GPKO and GKO+Gm26793 cells during EB differentiation.

(d-e) qPCR analyses of mesodermal (d) and PrE-related (e) genes on day 6 and day 8 of EB differentiation.

(f) The expression dynamics of *Fgf15* in indicated cell groups during EB differentiation.

(g) qPCR analyses of lineage-specific genes among respective cell types in EB differentiation.

(h) Transcriptomic correlation of XEN-D7 differentiation samples with decrease or increase in *Gm26793* and *Fgf15* expression.

All qPCR data are shown as means ± SEM. Two-way ANOVA analysis with Tukey's test is used in c, f; One-way ANOVA analysis with Tukey's test is used in d, e, g; ** p<0.01, *** p<0.001.

Fig. S6

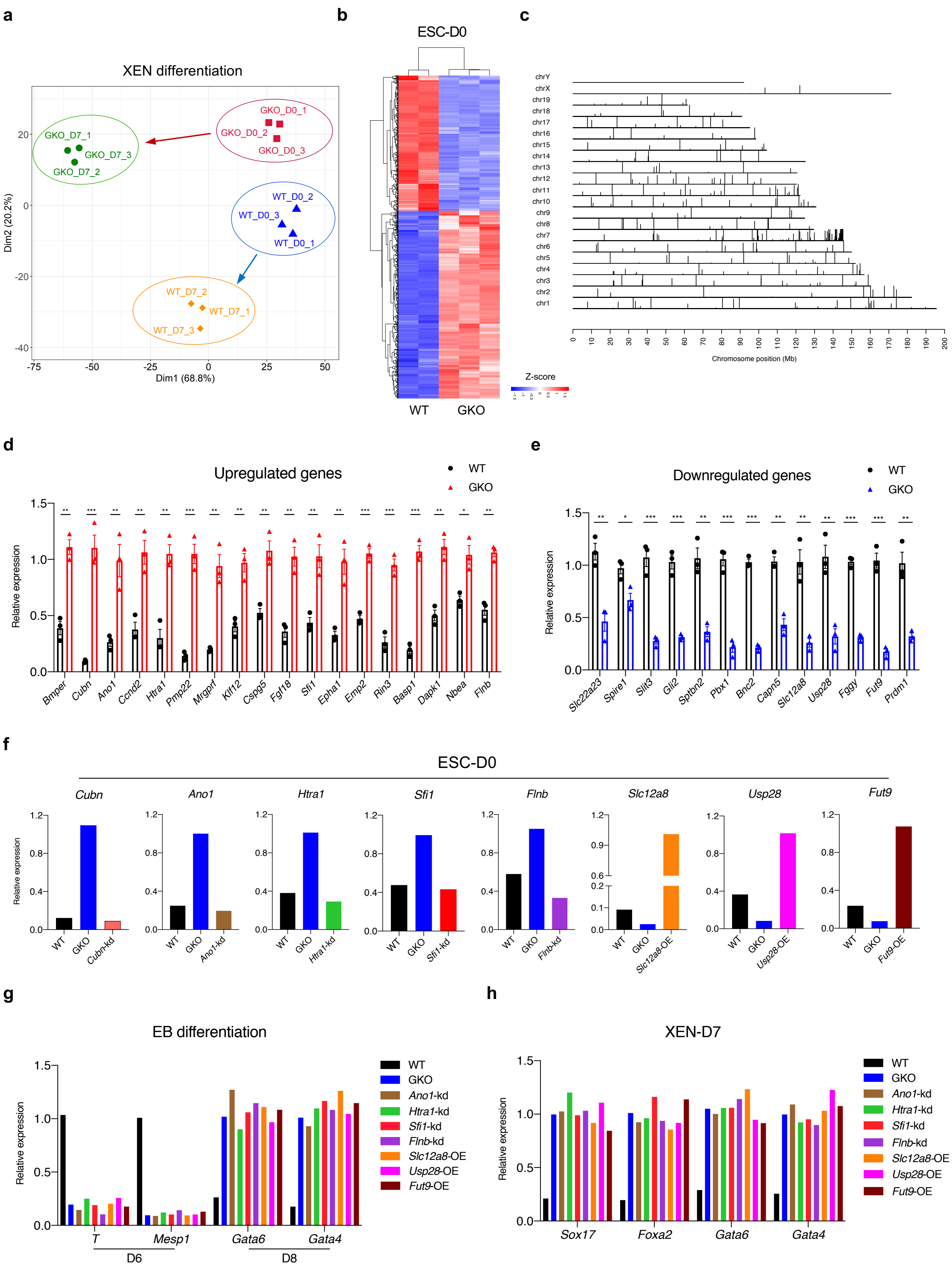

a
XEN differentiation
Dim1 (68.8%)
Dim2 (20.2%)
b
ESC-D0
WT
GKO
Z-score
c
Chromosome position (Mb)
d
Upregulated genes
e
Downregulated genes
Relative expression
f
ESC-D0
g
EB differentiation
D6
D8
h
XEN-D7

Fig. S6 Functional screening of genes regulated by *Gm26793* locus.

(a) PCA analyses of XEN differentiated samples showing the transcriptomic differences upon *Gm26793* knockout.

(b) Heatmap highlighting the direct transcriptomic comparison between WT and GKO mESCs.

(c) The genome-wide distribution of *Gm26793* interacting peaks captured by 4C-seq.

(d-e) qPCR validation of the top 31 genes in 39 selected candidates, in which 18 genes are up-regulated (d) and 13 genes are down-regulated (e) in GKO mESCs. Data are shown as means ± SEM. Student's t-test analysis; *$p<0.05$, ** $p<0.01$, *** $p<0.001$.

(f) Perturbation of candidate genes through knockdown or overexpression in GKO mESCs.

(g-h) qPCR analyses of lineage-specific genes in EB (g) and XEN (h) differentiation after expression perturbation of the candidate genes.

Fig. S7

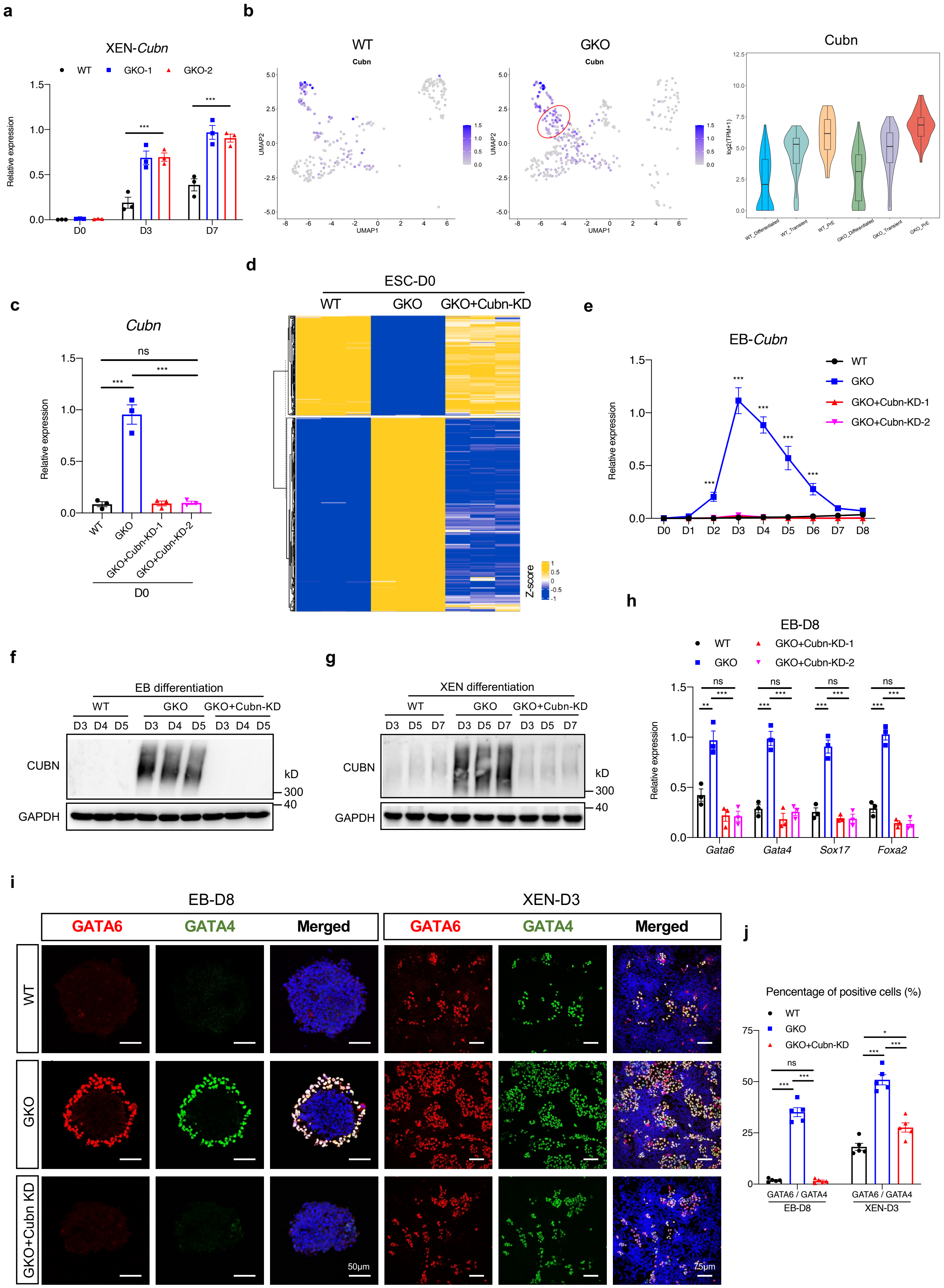

a
XEN-Cubn
WT GKO-1 GKO-2
Relative expression
D0 D3 D7
b
WT
GKO
Cubn
UMAP1
UMAP2
log2(TPM+1)
WT_Differentiated
WT_Transient
WT_PrE
GKO_Differentiated
GKO_Transient
GKO_PrE
c
Cubn
ns
WT
GKO
GKO+Cubn-KD-1
GKO+Cubn-KD-2
D0
d
ESC-D0
WT GKO GKO+Cubn-KD
Z-score
e
EB-Cubn
WT
GKO
GKO+Cubn-KD-1
GKO+Cubn-KD-2
D0 D1 D2 D3 D4 D5 D6 D7 D8
f
EB differentiation
WT GKO GKO+Cubn-KD
D3 D4 D5 D3 D4 D5 D3 D4 D5
CUBN
GAPDH
kD
300
40
g
XEN differentiation
WT GKO GKO+Cubn-KD
D3 D5 D7 D3 D5 D7 D3 D5 D7
CUBN
GAPDH
kD
300
40
h
EB-D8
WT GKO GKO+Cubn-KD-1 GKO+Cubn-KD-2
Gata6 Gata4 Sox17 Foxa2
i
EB-D8
XEN-D3
GATA6 GATA4 Merged
WT
GKO
GKO+Cubn KD
50μm
75μm
j
Pencentage of positive cells (%)
WT
GKO
GKO+Cubn-KD
GATA6 / GATA4
EB-D8
XEN-D3

Fig. S7 *Cubn* knockdown recovers the enhanced differentiation potential of PrE in GKO cells.

(a) Constant activation of *Cubn* in GKO cells during XEN differentiation.

(b) UMAP and violin plots showing the up-regulation of *Cubn* in transient epiblast and primitive endoderm cells.

(c) Bar plot showing the successful knockdown of *Cubn* expression in GKO+Cubn-KD mESCs.

(d) Heatmaps showing the transcriptomic recovery of GKO samples on ESC-D0 upon *Cubn* knockdown.

(e) Relative expression dynamics of *Cubn* in indicated cell groups during EB differentiation. (f-g) Protein expression changes of CUBN during EB (f) and XEN (g) differentiation upon *Cubn* knockdown.

(h) Reduced expression of PrE-related genes on day 8 of EB differentiation upon *Cubn* knockdown.

(i-j) Immunostaining images reporting the rescue of GATA6 and GATA4 expression pattern after *Cubn* knockdown in GKO cells. The specific differentiation system and timepoint for sampling are indicated at the top of each image. Scale bar, 50 μm and 75 μm. Quantification of the data is shown in (j).

All data are shown as means ± SEM. Two-way ANOVA analysis with Tukey's test is used in a, e; One-way ANOVA analysis with Tukey's test is used in c, h, j; *p<0.05, ** p<0.01, *** p<0.001.

Fig. S8

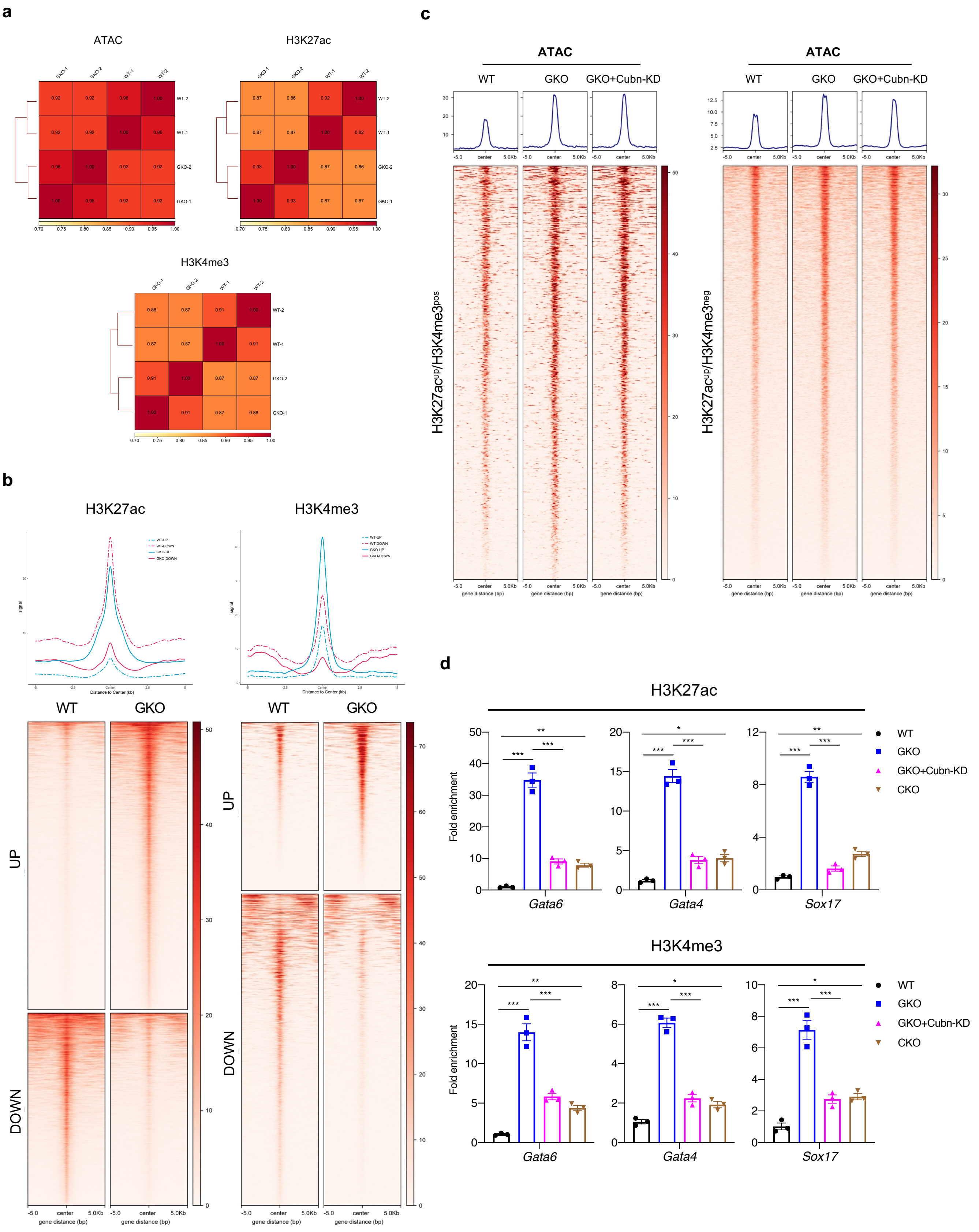

a
ATAC
H3K27ac
H3K4me3
GKO-1
GKO-2
WT-1
WT-2
b
H3K27ac
H3K4me3
WT-UP
WT-DOWN
GKO-UP
GKO-DOWN
Distance to Center (kb)
WT
GKO
UP
DOWN
gene distance (bp)
c
ATAC
WT
GKO
GKO+Cubn-KD
H3K27ac^up/H3K4me3^pos
H3K27ac^up/H3K4me3^neg
d
H3K27ac
H3K4me3
Fold enrichment
Gata6
Gata4
Sox17
WT
GKO
GKO+Cubn-KD
CKO

Fig. S8 ATAC-seq and ChIP-seq analysis.

(a) Spearman correlation of ATAC-seq, H3K27ac, as well as H3K4me3 signal in both WT and GKO replicates.

(b) Heat maps showing the global alteration of H3K27ac and H3K4me3 distribution in GKO cells in compare with WT mESCs. The averaged signal intensities for respective histone modification in each cell group are plotted at the top of each heatmap.

(c) Global distribution of ATAC-seq signal around selected genomic regions in WT, GKO, and GKO+Cubn-KD cells.

(d) ChIP-qPCR analyses validating the dynamic changes of H3K27ac and H3K4me3 enrichment around selected gene loci in indicated mESCs. Data are shown as means ± SEM. One-way ANOVA analysis with Tukey's test; *p<0.05, ** p<0.01, *** p<0.001.

Fig. S9

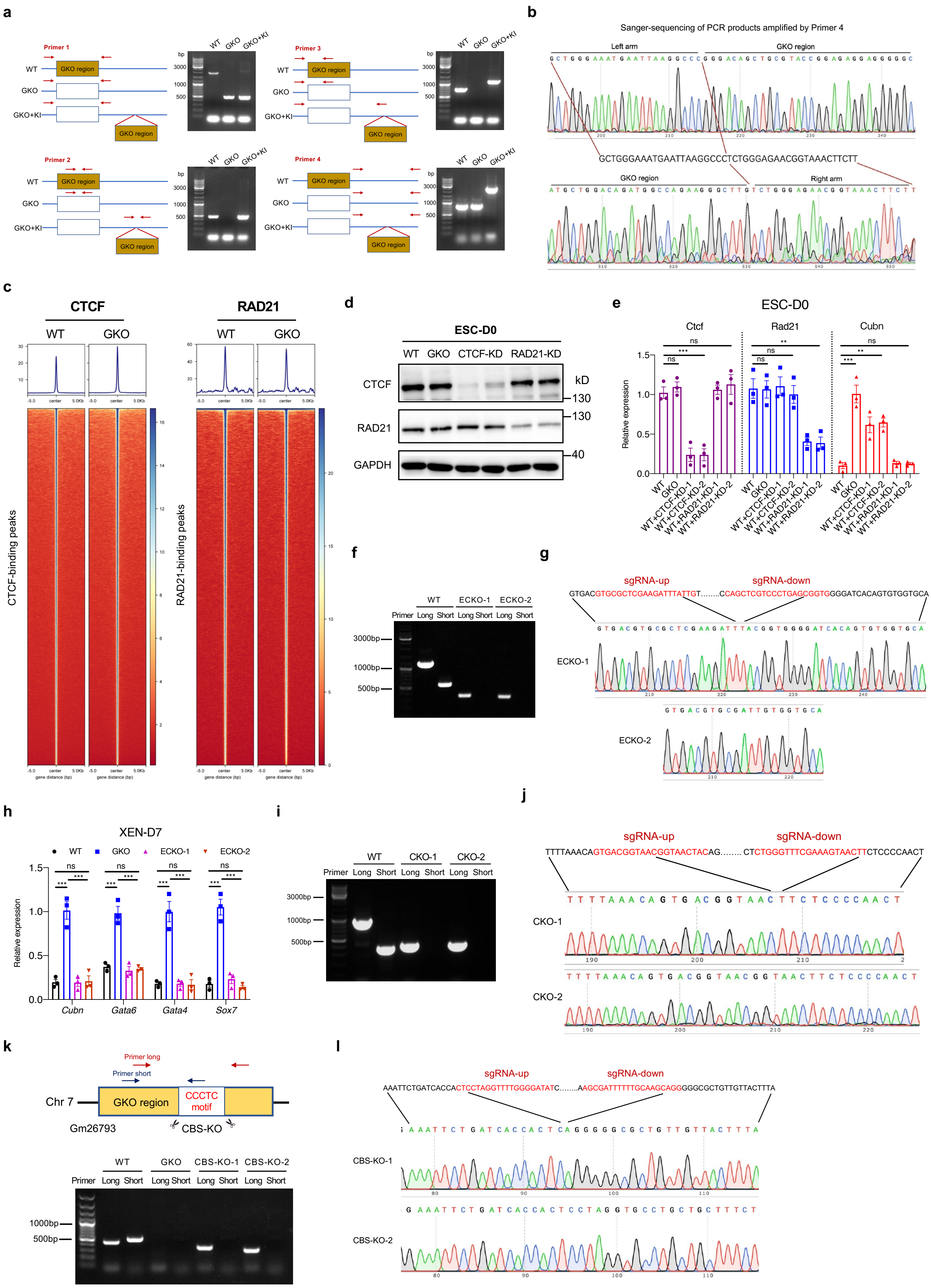

a
Primer 1
Primer 2
Primer 3
Primer 4
WT
GKO
GKO+KI
GKO region
bp
3000
1000
500
b
Sanger-sequencing of PCR products amplified by Primer 4
Left arm
GKO region
Right arm
GCTGGGAAATGAATTAAGGCCCTCTGGGAGAACGGTAAACTTCTT
c
CTCF
RAD21
CTCF-binding peaks
RAD21-binding peaks
gene distance (bp)
d
ESC-D0
WT GKO CTCF-KD RAD21-KD
CTCF
RAD21
GAPDH
kD
130
40
e
ESC-D0
Ctcf
Rad21
Cubn
Relative expression
WT
GKO
WT+CTCF-KD-1
WT+CTCF-KD-2
WT+RAD21-KD-1
WT+RAD21-KD-2
f
WT
ECKO-1
ECKO-2
Primer Long Short Long Short Long Short
3000bp
1000bp
500bp
g
sgRNA-up
sgRNA-down
GTGACGTGCGCTCGAAGATTTATTGT........CCAGCTCGTCCCTGAGCGGTGGGATCACAGTGTGGTGCA
ECKO-1
ECKO-2
h
XEN-D7
WT GKO ECKO-1 ECKO-2
Relative expression
Cubn
Gata6
Gata4
Sox7
i
WT
CKO-1
CKO-2
Primer Long Short Long Short Long Short
3000bp
1000bp
500bp
j
sgRNA-up
sgRNA-down
TTTTAAACAGTGACGGTAACGGTAACTACAG........ CTCTGGGTTTCGAAAGTAACTTCTCCCCAACT
CKO-1
CKO-2
k
Primer long
Primer short
Chr 7
GKO region
CCCTC motif
Gm26793
CBS-KO
WT GKO CBS-KO-1 CBS-KO-2
Primer Long Short Long Short Long Short Long Short
1000bp
500bp
l
sgRNA-up
sgRNA-down
AAATTCTGATCACCACTCCTAGGTTTTGGGGATATC.........AAGCGATTTTTTGCAAGCAGGGGCGCTGTTGTTACTTTA
CBS-KO-1
CBS-KO-2

Fig. S9 Establishment of CTCF binding site knock-in and kncockout mESCs.
(a) DNA electrophoresis showing the genotyping PCR results of GKO+KI mESCs.
(b) Sanger sequencing of PCR products amplified by Primer 4 demonstrating the successful re-insertion of GKO sequence in GKO mESCs.
(c) Heatmaps showing global CTCF and RAD21 binding signals in WT and GKO mESCs.
(d) Western blotting result reporting the decreased protein expression of CTCF and RAD21.
(e) Reduced expression of CTCF activates the transcription of *Cubn* in mESCs.
(f-g) DNA electrophoresis showing the efficient removal of ECKO locus in mESCs. The acquired truncated DNA sequences are determined by sanger sequencing (g).
(h) Bar plot showing the relative expression level of PrE-related marker genes in WT, GKO, and ECKO XEN.
(i-j) DNA electrophoresis showing the efficient removal of CKO locus in mESCs. The acquired truncated DNA sequences are determined by sanger sequencing (j).
(k-l) DNA electrophoresis showing the specific knockout of CTCF binding site within GKO region (CBS-KO). The acquired truncated DNA sequences are determined by sanger sequencing (l).
All qPCR data are shown as means ± SEM. One-way ANOVA analysis with Tukey's test is used in e, h; ** p<0.01, *** p<0.001.

Fig. S10

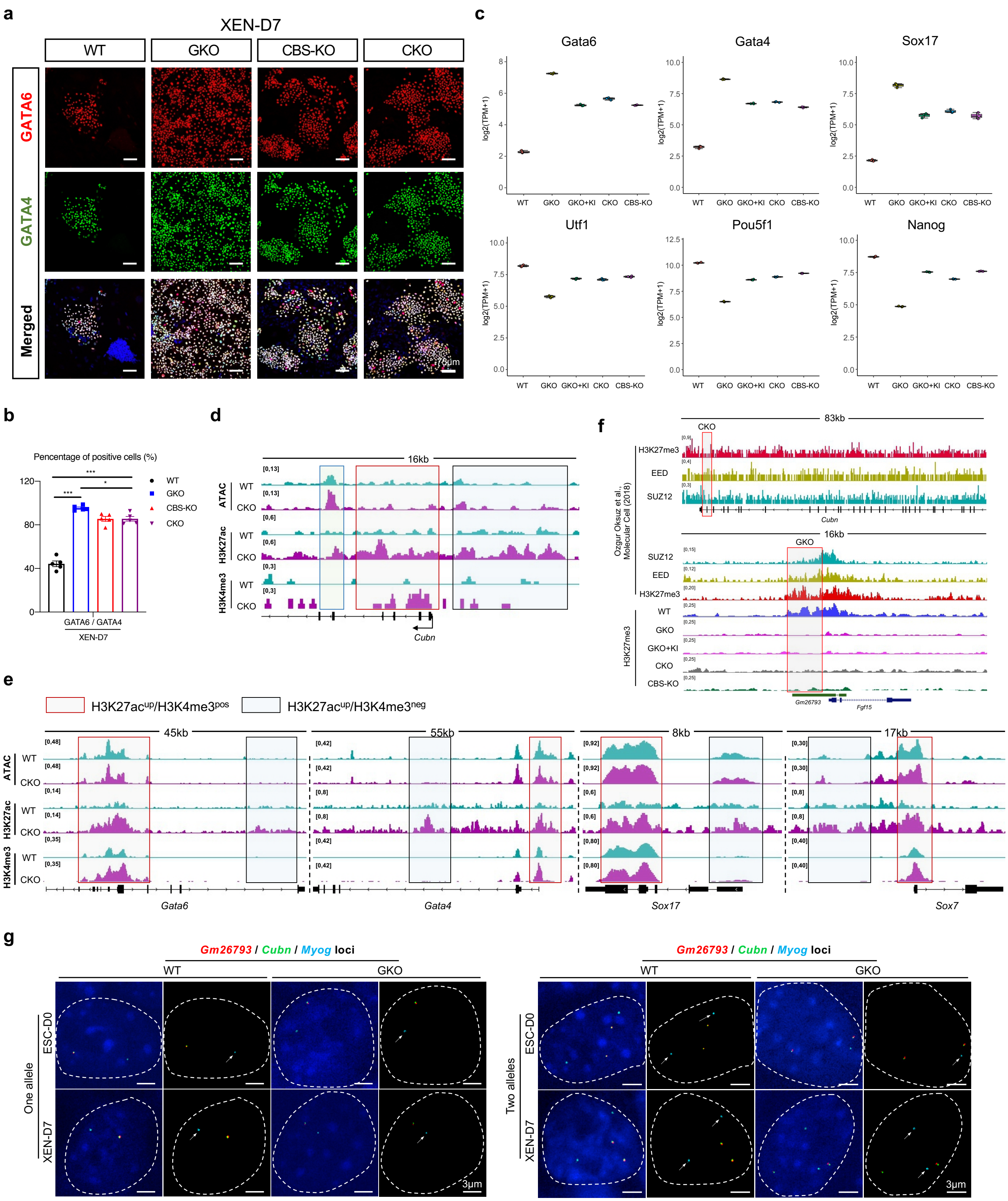

a
XEN-D7
WT
GKO
CBS-KO
CKO
GATA6
GATA4
Merged
75μm
b
Pencentage of positive cells (%)
GATA6 / GATA4
XEN-D7
c
Gata6
Gata4
Sox17
Utf1
Pou5f1
Nanog
log2(TPM+1)
d
16kb
ATAC
H3K27ac
H3K4me3
Cubn
e
H3K27ac^up/H3K4me3^pos
H3K27ac^up/H3K4me3^neg
Gata6
Gata4
Sox17
Sox7
f
83kb
Ozgur Oksuz et al., Molecular Cell (2018)
H3K27me3
EED
SUZ12
Cubn
16kb
GKO+KI
CBS-KO
Gm26793
Fgf15
g
Gm26793 / Cubn / Myog loci
One allele
Two alleles
ESC-D0
XEN-D7
3μm

Fig. S10 Functional validation of CTCF binding site in the formation of *Gm26793-Cubn* inter-chromosomal interaction.

(a-b) Representative images displaying the expression of GATA6 and GATA4 protein in XEN differentiated from WT, GKO, CBS-KO and CKO mESCs. Statistical analyses of fluorescent signal are summarized in (b) as bar plot. Scale bar, 75 μm. Data are shown as means ± SEM; One-way ANOVA analysis with Tukey's test; *p<0.05, *** p<0.001.

(c) Expression recovery of representative GKO-Up and -Down genes in indicated cell groups.

(d-e) Genome browser view showing the dynamics of chromatin accessibility, H3K27ac and H3K4me3 signals around *Cubn* locus (d) and PrE-related gene regions (e) in WT and CKO mESCs.

(f) Genome browser view demonstrating the distribution of SUZ12, EED and H3K27me3 binding signals around the *Gm26793* and *Cubn* loci in WT, GKO, GKO+KI, CKO and CBS-KO mESCs. Grey boxes represent the regions which were subjected to genomic knockout in WT cells.

(g) Representative images confirming the spatial separation of the *Myog* locus from the *Gm26793* and *Cubn* loci on ESC-D0 and XEN-D7. Scale bar, 3 μm.
